# Directional sorting of exciton emissions from twisted WS_2_/WSe_2_ hetero-bilayers using self-coupled photonic crystal resonances

**DOI:** 10.1126/sciadv.adu4968

**Published:** 2025-04-25

**Authors:** Yuhua Chen, Meng Xia, Jiaxin Zhou, Yuefeng Wang, Di Huang, Xingwang Zhang

**Affiliations:** ^1^ Suzhou Institute of Nano-Tech and Nano-Bionics (SINANO), Chinese Academy of Sciences (CAS), Suzhou, Jiangsu 215123, People’s Republic of China.; ^2^ School of Nano-Tech and Nano-Bionics, University of Science and Technology of China, Hefei, Anhui 230026, People’s Republic of China.; ^3^ Nano Science and Technology Institute, University of Science and Technology of China, Suzhou, Jiangsu 215123, People’s Republic of China.

## Abstract

Recent advances in two-dimensional semiconductor hetero-bilayers have revealed that the stacking angle between adjacent layers provides an additional degree of freedom to tune exciton states, enabling fascinating twist-angle–dependent photoluminescence. To control exciton emission properties, hetero-bilayers are usually integrated with photonic nanostructures, however, in which the contact interfaces can result in substantial luminescence suppression. To overcome this fundamental issue, we herein directly pattern photonic crystal (PhC) nanostructures in free-standing WS_2_/WSe_2_ hetero-bilayers to avoid the involvement of contact interfaces. Such PhC nanostructured WS_2_/WSe_2_ hetero-bilayers not only provide new exciton states but also offer guided mode resonances that can self-couple to excitons to enable light manipulation at the atomic thickness scale. Moreover, leveraging the unique momentum dispersion of guided mode resonances, exciton emissions are selectively excited and separated in the energy-momentum space. Our results may provide an important direction to unfold the prospects of emerging exciton states in the moiré heterostructures.

## Teaser

Directional sorting of exciton emissions is enabled by photonic crystal resonances in free-standing twisted WS_2_/WSe_2_ bilayers.

## INTRODUCTION

In recent years, van der Waals (vdW) heterostructures comprising a variety of two-dimensional (2D) layered transition metal dichalcogenides (TMDs) have emerged as novel atomically thin semiconductors, which not only combine the respective material functionalities but also acquire new characteristics through proximity interactions across interfaces (*1*–*3*). On account of the weak vdW bonding between neighboring layers of TMDs, monolayer (1L) TMDs can be isolated and stacked vertically to form synthetic heterostructures (*4*). Because of the weak vdW interaction between adjacent layers, new exciton states that are closely correlated to the interlayer coupling arise and coexist with the original intra-layer exciton states inside the constituent layers (*5*–*7*). Therefore, upon high-energy optical excitation, multiple exciton states can simultaneously emit photons with different colors. Unlike conventional semiconductor heterostructures that are limited by epitaxial growth, the constituent layers in TMDs heterostructures can have arbitrary lattice mismatch and twist angle, both of which can tailor the interlayer coupling (*8*). Therefore, for a hetero-bilayer with fixed TMDs components, the twist angle between the two layers provides an additional degree of freedom to tune the material properties, enabling fascinating twist angle–dependent multicolor luminescence (*5*, *9*, *10*).

On the other hand, the control of light emission direction is key for many applications that use light or photons as the media for carrying and/or processing information (*11*–*15*). In general, realizing directional luminescence requires a photonic resonance to interact with the luminescent media in the near field and then route the light emission to the far field (*16*). However, owing to the lack of photonic resonances in TMDs themselves, it is inevitable to rely on external optical resonators to provide photonic resonances to interact with excitons in TMDs (*17*–*19*). For this reason, various artificial nanophotonic structures, such as nano-antennas and photonic crystals (PhCs), have been used to integrate with TMDs to tailor the direction of exciton emission (*20*–*23*). In particular, the highly dispersive guided mode resonances in dielectric PhC slabs integrated with TMDs can be harnessed to achieve directional exciton emission with distinctive wavelength dependence (*21*, *22*, *24*). Although these external photonic resonances can strongly interact with excitons using intra-cavity electric field enhancement or confined evanescent field, the interface of the integrated dielectric materials may introduce n-type doping and defect-assisted nonradiative exciton recombination in TMDs, leading to luminescence suppression (*21*, *25*). What is more, for TMDs hetero-bilayers, the integrated dielectric materials cause strong dielectric screening effects that substantially reduce the binding energies of interlayer excitons, which in turn severely suppress the luminescence of interlayer excitons (*26*). Therefore, it is natural to ask whether there is a way to directly introduce photonic resonances in free-standing twisted hetero-bilayers themselves such that we can not only achieve efficient interlayer exciton emissions by suspending the materials in the air but also sort the emissions of multiple exciton states through their directionalities.

**Fig. 1.**
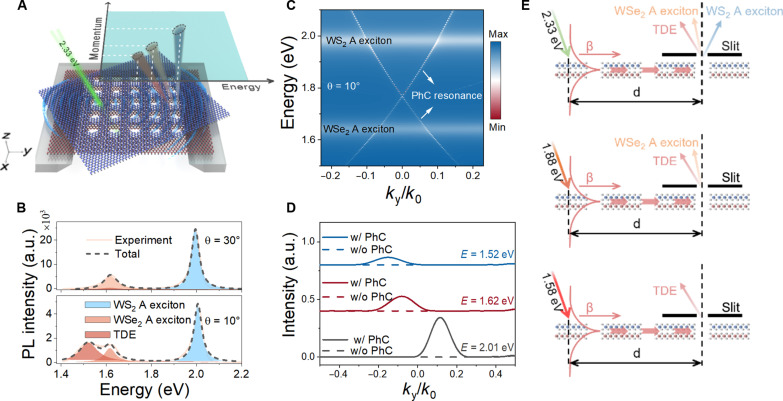
Conceptual illustration for the directional sorting of exciton emissions from free-standing PhC-nanostructured WS_2_/WSe_2_ hetero-bilayers. (**A**) Light emitted by excitons in the twisted WS_2_/WSe_2_ hetero-bilayer with a stacking angle of θ is coupled to the guided mode resonances coexisting in the free-standing PhC-nanostructured WS_2_/WSe_2_ hetero-bilayers and then sorted in the energy-momentum space. (**B**) PL spectra for free-standing PhC nanostructured WS_2_/WSe_2_ hetero-bilayers with a stacking angle of 30° (top) and 10° (bottom), respectively. The shaded regions represent the curves fitted by multiple Lorentz functions, from which we can extract the contributions of WS_2_ A exciton, WSe_2_ A exciton, and TDE from the spectra. The dashed lines denote the sum of the multiple Lorentz fittings. (**C**) Simulated momentum-resolved transmission spectra for a free-standing PhC-nanostructured WS_2_/WSe_2_ hetero-bilayer with a twist angle of 10°, in which WS_2_ A exciton, WSe_2_ A exciton, and PhC-guided mode resonances can be observed. (**D**) Simulation results for momentum-resolved exciton emission for a free-standing WS_2_/WSe_2_ hetero-bilayer with a twist angle of 10° in the presence (w/ PhC) and in the absence (w/o PhC) of PhC nanostructures. *E* = 2.01, 1.62, and 1.52 eV indicate the peak energies for WS_2_ A exciton, WSe_2_ A exciton, and TDE, respectively. (**E**) Schematic side views (i.e., *yz* plane) of free-standing PhC nanostructured WS_2_/WSe_2_ hetero-bilayers under different excitation energies. A slit is placed at a distance (d) away from the excitation spot as a spatial filter. Excitons can be selectively excited according to the excitation laser energy. Only the emitted light that couples to the guided mode resonance and reaches the slit position can be sorted by the emission directionality. β denotes the propagation constant of the guided mode resonance.

To this end, we present the experimental demonstration for the directional sorting of exciton emissions from twisted tungsten disulfide/tungsten diselenide (WS_2_/WSe_2_) hetero-bilayers using self-coupled PhC-guided mode resonances (Fig. 1A). By suspending WS_2_/WSe_2_ hetero-bilayers in the air by using a silicon frame, we can essentially eliminate potential luminescence suppression effects suffered from the dielectric contact interface. As can be seen in Fig. 1B, strong emissions from the intra-layer A excitons in the constituting WS_2_ and WSe_2_ layers are observed in the photoluminescence (PL) spectra. In addition to this, the interlayer coupling engenders a new exciton state with remarkable twist-angle dependence [i.e., twist angle–dependent exciton (TDE)]. In the meantime, PhC nanostructures with a square lattice of air holes are constructed directly out of the free-standing WS_2_/WSe_2_ hetero-bilayers to introduce guided mode resonances (Fig. 1C) (*24*, *27*, *28*). Hence, the free-standing PhC-nanostructured WS_2_/WSe_2_ hetero-bilayers not only provide new exciton states but also offer guided mode resonances that can self-couple to excitons to enable light manipulation at the atomic thickness scale. Such a self-coupling scheme is critical to manipulate the exciton emissions in the free-standing atomically thin TMDs because there are no external dielectric optical resonators to provide optical resonances in the suspended hetero-bilayers. Furthermore, guided mode resonances in the free-standing PhC nanostructured WS_2_/WSe_2_ hetero-bilayers have unique momentum dispersion properties so that both the intra-layer and interlayer driven exciton emissions can be selectively excited by choosing appropriate pump laser energy and resonantly sorted in the energy-momentum space (Fig. 1, D and E).

## RESULTS

**Fig. 2.**
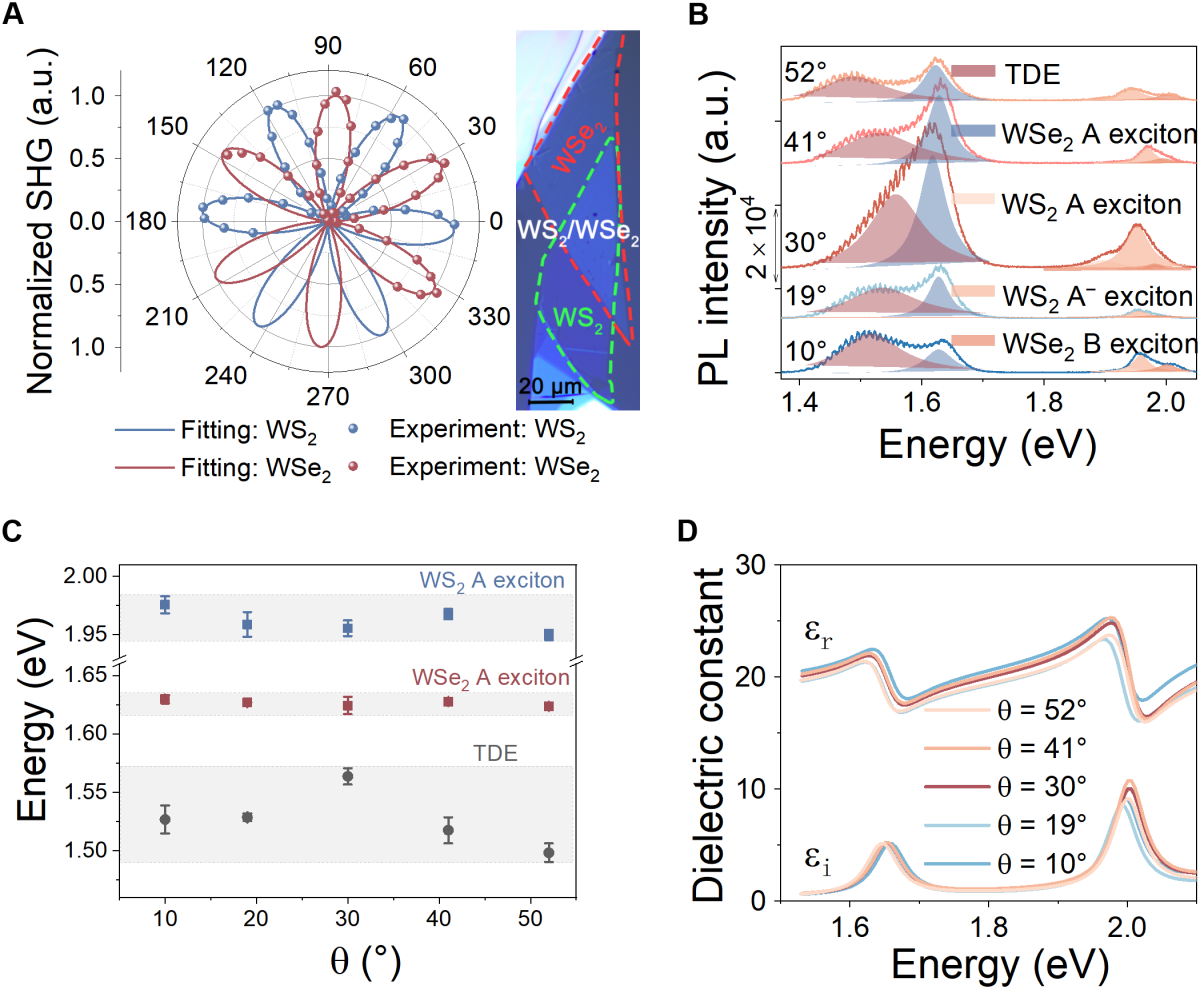
Characterization of twisted WS_2_/WSe_2_ hetero-bilayers. (**A**) Polarization-dependent SHG patterns for the WS_2_ and WSe_2_ 1Ls. The stacking angle between WS_2_ and WSe_2_ 1Ls is determined to be 30° by their SHG patterns. Right, optical microscopy image of vertically stacked WS_2_ and WSe_2_ 1Ls. The red and green dashed curves denote the boundaries for WS_2_ and WSe_2_ 1Ls, respectively. Scale bar, 20 μm. (**B**) PL spectra for twisted WS_2_/WSe_2_ hetero-bilayers with stacking angles of 10°, 19°, 30°, 41°, and 52°. The spectra are fitted by multiple Lorentz functions to extract the contributions of various excitons in the hetero-bilayers. The PL spectra are stacked vertically with different PL intensity bias. (**C**) Extracted energies for WS_2_ A exciton, WSe_2_ A exciton, and TDE from (B) as a function of stacking angle θ. The error bars are obtained by multiple measurements. (**D**) Measured dielectric constants for WS_2_/WSe_2_ hetero-bilayers with different stacking angles. ε_r_ and ε_i_ represent the real and imaginary parts of the dielectric constant, respectively.

To get insights into the directional sorting of the exciton emissions from PhC-nanostructured WS_2_/WSe_2_ hetero-bilayers, it is valuable to begin with the study of optical properties of twisted WS_2_/WSe_2_ hetero-bilayers. The twist angle between the two layers offers a degree of freedom to tune the interlayer coupling, which can be harnessed to manipulate exciton emissions in WS_2_/WSe_2_ hetero-bilayers. With this in mind, we first characterize the twist angle–dependent PL spectra of WS_2_/WSe_2_ hetero-bilayers. For this purpose, all the 1L TMDs used are first mechanically exfoliated from bulk crystals and stacked together on fused silica substrates using a dry-transfer method (Fig. 2A; see Methods and note S1 for more details on the sample fabrication). The stacking angle between WS_2_ and WSe_2_ can be determined optically using polarization-dependent second harmonic generation (SHG) measurement (Fig. 2A; see Methods and note S2 for more details on the polarization dependent SHG characterization). Because of their intrinsic broken inversion symmetries, both 1L WS_2_ and 1L WSe_2_ display sixfold SHG patterns, from which we can determine the relative twist angle between the two layers (Fig. 2A). To comprehensively study the twist angle–dependent PL spectra, Fig. 2B shows the PL spectra for WS_2_/WSe_2_ hetero-bilayers with different stacking angles (θ = 10°, 19°, 30°, 41°, and 52°), which reveal prominent twist-angle dependence. We then use multiple Lorentz functions to fit the PL spectra to analyze the contribution of each exciton. Apart from WS_2_ and WSe_2_ excitons, there is another exciton emerging around 1.52 eV, whose emission energy is highly dependent on the stacking angle. Such a TDE is attributed to the interlayer coupling between 1L WS_2_ and 1L WSe_2_ (*9*, *10*, *29*). To quantitatively evaluate the twist-angle dependence of the emission energies for these excitons, we can extract the energies for WS_2_ A exciton, WSe_2_ A exciton, and TDE from the Lorentz fitting (Fig. 2C). The peak energies of WS_2_ and WSe_2_ A excitons only have very weak twist-angle dependence. In contrast, the energy of TDE is strongly correlated to the stacking angles, which offers a way to tune the emission energy of TDE by controlling the stacking angle between the two layers. We note that the PL of hetero-bilayers is usually quenched compared with the 1L exciton emission due to the ultrafast interlayer charge transfer (*30*) and dielectric screening (*31*, *32*). However, compared with the few-layer WS_2_ and WSe_2_, the hetero-bilayers show much stronger PL intensity (note S3). Therefore, by stacking 1L WS_2_ and WSe_2_ together, we can obtain more efficient luminescence and higher tunability than their few-layer counterparts.

To facilitate the design of PhC nanostructures, we need to measure the twist angle–dependent dielectric constants of WS_2_/WSe_2_ hetero-bilayers. For this purpose, we measure the reflection spectra for WS_2_/WSe_2_ hetero-bilayers with different stacking angles and then extract the dielectric constants from their reflection spectra using Kramers-Kronig constrained variational analysis based on the Lorentz model (note S4) (*24*, *25*, *33*). From the imaginary part of dielectric constants (ε_*i*_), we note that there are two strong exciton resonances in WS_2_/WSe_2_ hetero-bilayers with the energy below 2.1 eV, corresponding to the A excitons in the WS_2_ and WSe_2_ layers, respectively (Fig. 2D). Since the energies for WS_2_ and WSe_2_ excitons have very weak twist-angle dependence (Fig. 2C), the corresponding dielectric constants near these exciton energies have no obvious differences at different stacking angles (Fig. 2D). On the other hand, it is well known that the exciton oscillator strength for TDE is two orders of magnitude smaller than that of the intra-layer excitons due to the reduced transition dipoles (*34*–*36*). Thus, the dielectric constants have no visible features associated with TDE, although we can observe strong TDE emission in the PL spectra.

**Fig. 3.**
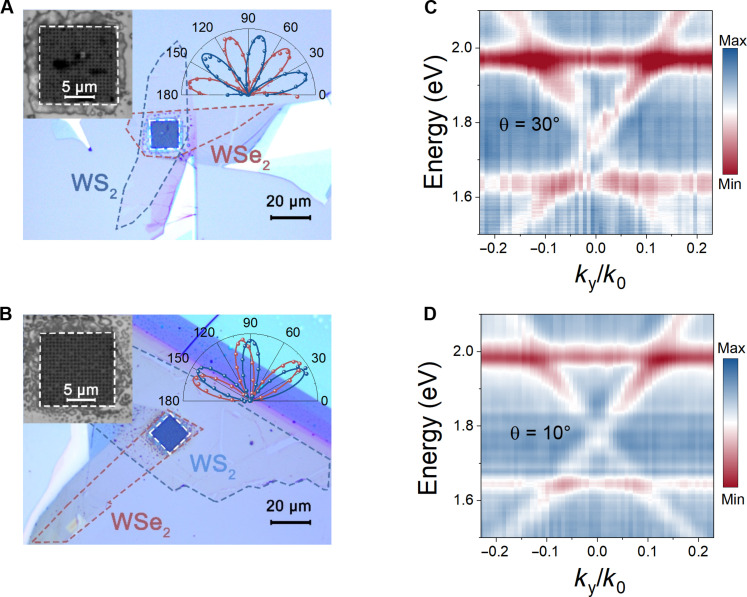
Characterization of free-standing PhC-nanostructured WS_2_/WSe_2_ hetero-bilayers. Optical microscopy images for free-standing PhC-nanostructured WS_2_/WSe_2_ hetero-bilayers with a stacking angle of 30° (**A**) and 10° (**B**), respectively. Scale bars, 20 μm. The dashed white squares indicate the suspended region. Polarization-dependent SHG patterns for WS_2_ (blue) and WSe_2_ (brown) are overlaid to determine the orientations for 1L crystals. For the sample in (A), the lattice constant is about 705 nm and radius of air holes is about 185 nm. For the sample in (B), the lattice constant is about 700 nm and radius of air holes is about 240 nm. Measured momentum-resolved transmission spectra for free-standing PhC-nanostructured WS_2_/WSe_2_ hetero-bilayers with a stacking angle of 30° (**C**) and 10° (**D**), respectively.

We then continue to fabricate and characterize free-standing PhC-nanostructured WS_2_/WSe_2_ hetero-bilayers in our experiment. To this end, we directly pattern PhC nanostructures in the free-standing WS_2_/WSe_2_ hetero-bilayers (see Methods and note S1 for more details on the sample fabrication). Figure 3 (A and B) show the optical microscopy images for two fabricated free-standing PhC nanostructured WS_2_/WSe_2_ hetero-bilayers with stacking angles of 30° and 10°, respectively. The stacking angle for each sample is determined by the polarization-dependent SHG measurement. To show the momentum dispersion of the PhC-nanostructured WS_2_/WSe_2_ hetero-bilayers, we have measured their momentum-resolved transmission spectra. As shown in Fig. 3 (C and D), in addition to the characteristic material-dependent WS_2_ and WSe_2_ excitonic features, momentum-dispersive guided mode resonances emerge in the measured transmission spectra as new structure-dependent spectral features. These resonances arise because the guided modes that are previously below the light line are folded above the light line in the first Brillouin zone, becoming guided mode resonances that can be excited by free space incidence (*37*, *38*). On the other hand, we also notice that the measured guided mode resonances match the simulated spectra very well except for much broader linewidth (note S5). Such a pronounced linewidth broadening is attributed to the fabrication imperfections, which introduce additional nonradiative losses in the PhC nanostructures (*24*). Meanwhile, we cannot see any anti-crossing phenomena in Fig. 3 (C and D). According to our theoretical analysis by using the temporal coupled-mode theory, we can conclude that the exciton-photon coupling in the free-standing PhC nanostructured WS_2_/WSe_2_ hetero-bilayers is in the weak coupling regime (note S6). In addition, since the dielectric constants of WS_2_/WSe_2_ hetero-bilayers for different stacking angles are similar, the momentum-resolved transmission spectra of both samples are almost independent of the stacking angle (note S5).

The coexistence of photonic resonances and excitons in WS_2_/WSe_2_ hetero-bilayers ensures the maximized spatial overlap between photons and excitons so that the manipulation of exciton emissions can be resonantly enhanced by the photonic resonances with this self-coupling scheme. The presence of guided mode resonances in the free-standing WS_2_/WSe_2_ hetero-bilayers can not only enhance the Purcell factors but also extraction rates of excitons, which can resonantly enhance the exciton emission at the atomic thickness scale (note S6). Furthermore, because of the momentum dispersion of PhC nanostructures, the photonic resonances can spectrally overlap with each type of excitons with a specific in-plane momentum. As can be seen in Fig. 3 (C and D), there is one PhC resonance that linearly blue-shifts in the spectral range of 1.75 ~ 2.1 eV with the increase of in-plane momentum. At *k*_*y*_/*k*_0_ ≈ ±0.11, the PhC resonance spectrally overlaps with the WS_2_ exciton. On the other hand, there is another PhC resonance that linearly red-shifts in the spectral range of 1.5 ~ 1.75 eV with the increase of *k*_*y*_/*k*_0_, which spectrally overlaps with the WSe_2_ exciton at *k*_*y*_/*k*_0_ ≈ ±0.08. Hence, these two PhC resonances can respectively interact with excitons in WS_2_/WSe_2_ PhC nanostructures at different in-plane momentums. Consequently, excitons in hetero-bilayers can acquire certain in-plane momentums depending on their energies and then emit to specific locations in the energy-momentum space (note S7).

**Fig. 4.**
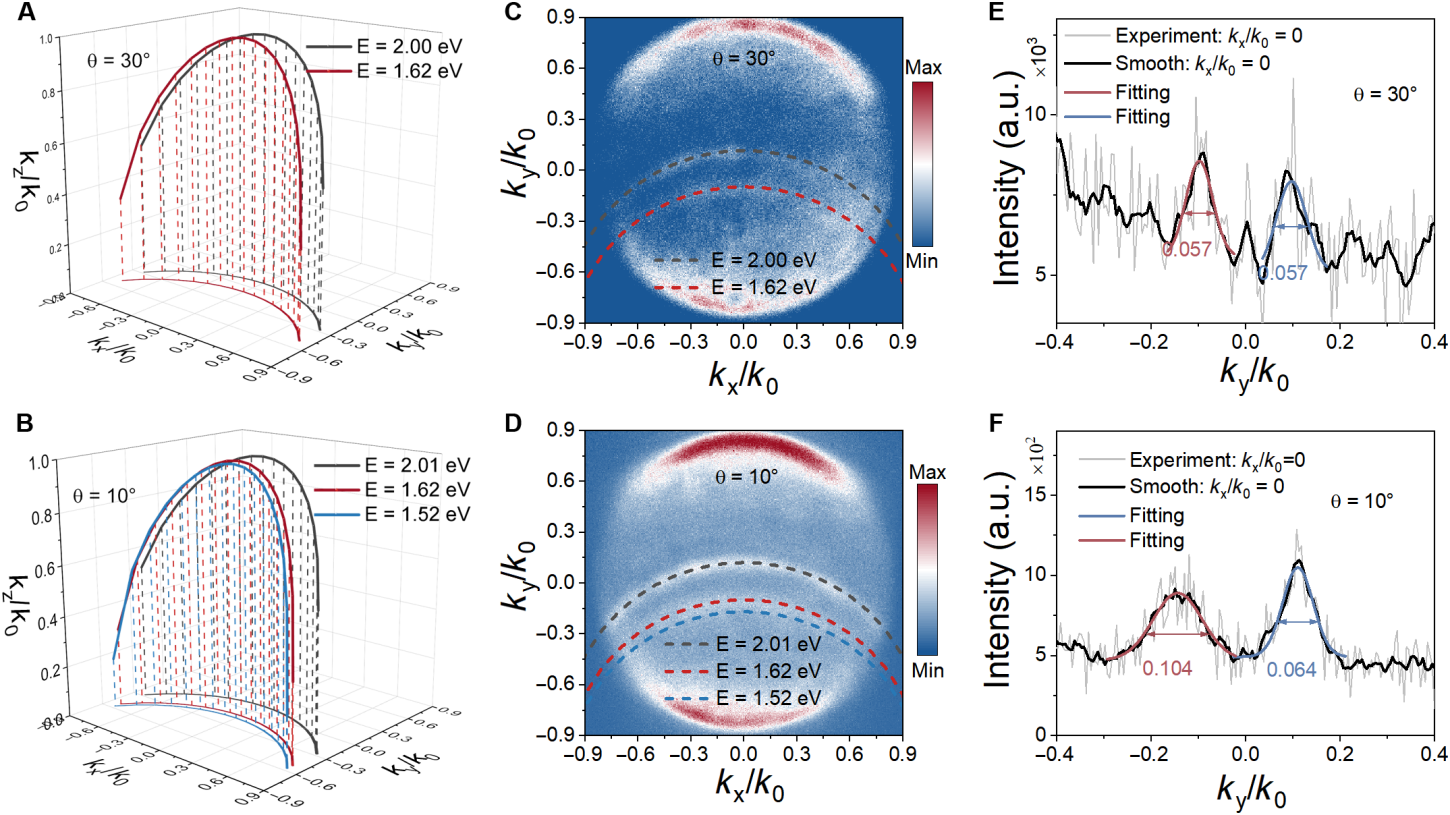
Sorting of exciton emissions from free-standing PhC-nanostructured WS_2_/WSe_2_ hetero-bilayers in the momentum space. Calculated equi-frequency contours at peak energies of excitons in free-standing PhC-nanostructured WS_2_/WSe_2_ hetero-bilayers stacked at 30° (**A**) and 10° (**B**). Measured Fourier plane image of exciton emissions from free-standing PhC-nanostructured WS_2_/WSe_2_ hetero-bilayers stacked at 30° (**C**) and 10° (**D**). The dashed curves are the calculated equi-frequency contours at peak energies of excitons in free-standing PhC-nanostructured WS_2_/WSe_2_ hetero-bilayers. Vertical linecuts at *k*_*x*_/*k*_0_ = 0 in (C) (**E**) and (D) (**F**). The emission peaks are fitted by Gaussian profiles to extract the linewidths.

Next, we proceed to demonstrate the directional sorting of the exciton emissions from the free-standing PhC-nanostructured WS_2_/WSe_2_ hetero-bilayers. As schematically shown in Fig. 1A, the exciton emission can be optically excited from the twisted WS_2_/WSe_2_ layers, which in turn couple to the guided mode resonances in the free-standing WS_2_/WSe_2_ PhC nanostructures via near-field interaction. The presence of PhC nanostructures in the free-standing WS_2_/WSe_2_ hetero-bilayers introduces an extra in-plane momentum, which enables momentum matching condition between the wave vector inside the hetero-bilayer and the free-space wave vector. Consequently, leveraging the unique momentum dispersion property of atomically thin PhC nanostructures, light emitted from excitons is coupled to the free-space with an energy-dependent position in the momentum space, which is determined by the momentum matching condition (note S7). To this end, the momentum matching condition between the guided mode resonance and the in-plane component of the free-space wave vector (*k*_0_) can be expressed as

$${\left(n_{\mathrm{eff}}\right)}^{2}={\left(\frac{k_{x}}{k_{0}}+m\frac{2\pi }{\Lambda k_{0}}\right)}^{2}+{\left(\frac{k_{y}}{k_{0}}+n\frac{2\pi }{\Lambda k_{0}}\right)}^{2},\;m,n=0,1,2\ldots$$ (1)

where *n*_*eff*_ is the effective index of the guided mode resonance, Λ is the PhC period, and *m* and *n* are the orders of the modes (note S7). The calculated equi-frequency contours (*m* = 0 and *n* = 1) at the exciton resonances in the twisted WS_2_/WSe_2_ PhC nanostructures with the stacking angles of 30° and 10° are plotted in Fig. 4 (A and B, respectively). In our experiment, we use a 2.33-eV continuous wave laser to excite the exciton emissions and an objective with a numerical aperture (NA) of 0.9 to collect the diffracted light passing through a slit (6 μm away from the excitation spot) and then project the Fourier plane of the luminescent WS_2_/WSe_2_ PhC nanostructures onto a camera to measure the equi-frequency contours. For WS_2_/WSe_2_ PhC nanostructures with a stacking angle of 30°, the energy of TDE emission is quite close to that of WSe_2_ A exciton, leaving only two PL peaks in the PL spectra (Fig. 1C). In consequence, we observe two bright arcs in the Fourier plane of the WS_2_/WSe_2_ PhC nanostructures (Fig. 4C). By comparing with the calculated equi-frequency contours, we can conclude that the upper arc is associated with the WS_2_ A exciton at ~2.00 eV, while the lower arc is related to the WSe_2_ A exciton at ~1.62 eV and the overlapped TDE. By taking a vertical linecut at *k*_*x*_/*k*_0_ = 0 as shown in Fig. 4E, we can see that the light emission from the WS_2_/WSe_2_ PhC nanostructures is routed to two different directions at *k*_*y*_/*k*_0_ = −0.089 and 0.097, respectively. Regarding WS_2_/WSe_2_ PhC nanostructures with a stacking angle of 10°, the TDE emission peak is red-shifted to 1.52 eV in respect to the WSe_2_ A exciton (Fig. 1C). Hence, the lower bright arc at in the Fourier plane becomes broader because of the increased linewidth (Fig. 4D, ∆*k*_*y*_/*k*_0_). We then numerically fit the peaks in the vertical linecuts in Fig. 4 (E and F) to extract the momentum linewidth (∆*k*_*y*_/*k*_0_). Compared with the WS_2_/WSe_2_ PhC nanostructures with a stacking angle of 30°, the momentum linewidth for the lower arc is much broader for the WS_2_/WSe_2_ PhC nanostructures with a stacking angle of 10°.

**Fig. 5.**
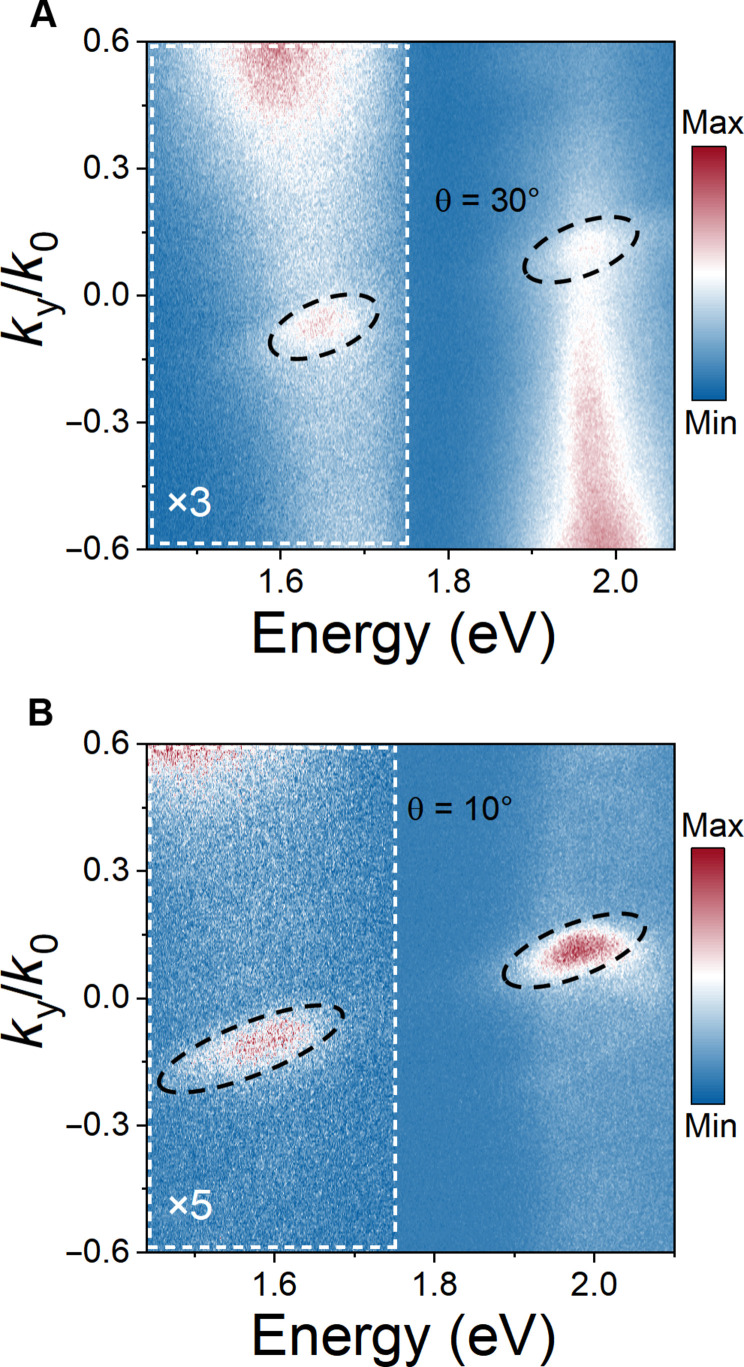
Sorting of exciton emissions from free-standing PhC-nanostructured WS_2_/WSe_2_ hetero-bilayers in the energy-momentum space. Measured momentum-resolved PL spectra for the free-standing PhC-nanostructured WS_2_/WSe_2_ hetero-bilayers stacked at 30° (**A**) and 10° (**B**). The dashed circles indicate the emission regions. The region between 1.45 and 1.75 eV is, respectively, magnified by three (A) and five (B) times for better view.

Although exciton emissions with different energies can be separated in the momentum space by the self-coupled guided mode resonances, the Fourier space images in Fig. 4 lose energy information. Bearing this in mind, we can simultaneously make use of both momentum and energy dimensions to sort the exciton emissions in twisted WS_2_/WSe_2_ PhC nanostructures. To this end, the Fourier plane of the luminescent WS_2_/WSe_2_ PhC nanostructures is diffracted by a grating before projected onto the camera. The momentum-resolved PL spectra in Fig. 5 (A and B) illustrate the sorting of the twist angle–dependent light emission from WS_2_/WSe_2_ hetero-bilayers in energy-momentum space. For better illustration, we use dashed circles to indicate the emission regions in the energy-momentum space. In this case, we can not only also show the in-plane momentums of emitted excitons but also reveal the corresponding emission energies. For both WS_2_/WSe_2_ PhC nanostructures, the forward emission spots (*k*_*y*_/*k*_0_ > 0) correspond to the WS_2_ A excitons around 2.0 eV, while the backward emission spots (*k*_*y*_/*k*_0_ < 0) are associated with the WSe_2_ A excitons and TDEs. In addition, compared with WS_2_/WSe_2_ PhC nanostructures with a stacking angle of 30°, in which the energy of TDE emission is almost overlapped with that of WSe_2_ A exciton, the sample with a stacking angle of 10° exhibits a broader emission spot in the energy range of 1.5 to 1.6 eV due to the existence of red-shifted TDE emission.

**Fig. 6.**
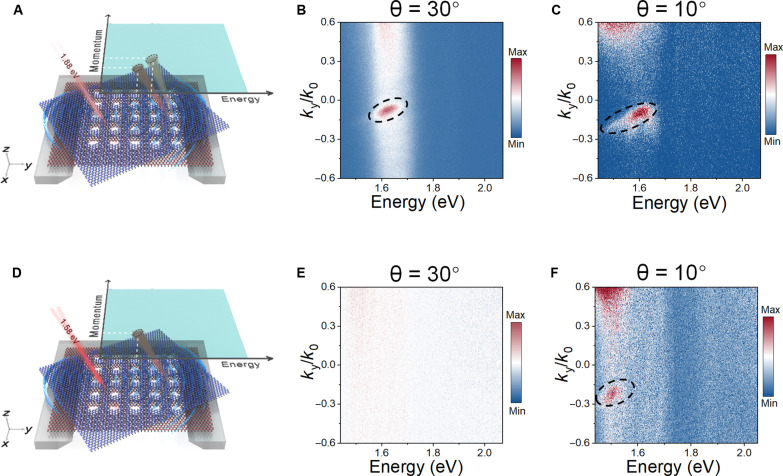
Selective sorting of exciton emissions from free-standing PhC-nanostructured WS_2_/WSe_2_ hetero-bilayers in the energy-momentum space. Schematic drawings for free-standing PhC-nanostructured WS_2_/WSe_2_ hetero-bilayers excited by 1.88 eV (**A**) and 1.58 eV (**D**) lasers. Measured momentum-resolved PL spectra for the free-standing PhC-nanostructured WS_2_/WSe_2_ hetero-bilayers stacked at 30° (**B**) and 10° (**C**) under 1.88-eV laser excitations. Measured momentum-resolved PL spectra for the free-standing PhC-nanostructured WS_2_/WSe_2_ hetero-bilayers stacked at 30° (**E**) and 10° (**F**) under 1.58-eV laser excitations. The dashed circles in [(B), (C), and (F)] indicate the emission regions.

As described above, the momentum dispersion of self-coupled guided mode resonances enables the sorting the exciton emissions in twisted WS_2_/WSe_2_ PhC nanostructures in the energy-momentum domain. Since these exciton states differ from each other in the emission energies, we can selectively excite and separate exciton emissions by using appropriate laser energy (note S8). With this in mind, we change the excitation laser energy from 2.33 to 1.88 eV (Fig. 6A). Such an excitation energy is below that of the WS_2_ A exciton but above those of the WSe_2_ A exciton and TDE. Therefore, by using 1.88-eV excitation, we can selectively eliminate the WS_2_ A exciton emission. As a result, for the WS_2_/WSe_2_ PhC nanostructure with a stacking angle of 30°, the bright spot that is previously observed around 2.0 eV in Fig. 5A disappears, leaving only an emission spot around 1.6 eV in Fig. 6B. Similarly, for the WS_2_/WSe_2_ PhC nanostructure with a stacking angle of 10°, the WSe_2_ A exciton and TDE emissions result in a wider spot in Fig. 6C, while the WS_2_ A exciton emission vanishes. By further reducing the excitation laser energy, it is possible to solely excite and isolate the TDE emission. To this end, we change the excitation laser energy to 1.58 eV (Fig. 6D), which is below those of WS_2_ A exciton (2.01 eV) and WSe_2_ A exciton (1.62 eV) while above that of TDE (1.52 eV) for the WS_2_/WSe_2_ hetero-bilayer with a stacking angle of 10° (Fig. 1B). Therefore, only TDE emission can be excited in the WS_2_/WSe_2_ PhC nanostructures. For this reason, we observe that there is only one bright spot around 1.52 eV, which indicates that only the TDE emission is excited in the energy-momentum domain in Fig. 6F. In contrast, for the WS_2_/WSe_2_ PhC nanostructure with a stacking angle of 30°, whose TDE emission energy (1.61 eV) is close to that of WSe_2_ A exciton and above that of the excitation laser, we cannot observe any PL signal in the energy-momentum domain in Fig. 6E.

## DISCUSSION

In conclusion, we have experimentally demonstrated the sorting of exciton emissions from free-standing WS_2_/WSe_2_ PhC nanostructures. Compared with other exciton emission control methods which inevitably require the integration of photonic nanostructures constructed by other materials, our method can essentially eliminate luminescence suppression effects suffered from the contact interfaces. In the meantime, the suspension of twisted WS_2_/WSe_2_ hetero-bilayers in the air reduces their environment dielectric constants to 1.0, which notably reduces the dielectric screening effect, leading to remarkably enhanced binding energies of excitons (note S9). In addition, leveraging the momentum dispersion property of PhC nanostructures, exciton emissions in twisted WS_2_/WSe_2_ PhC nanostructures can be selectively excited and separated in the energy-momentum space. Such an exciton emission manipulation method at the atomic thickness scale is enabled by the self-coupled guided mode resonances which coexist with excitons in the free-standing WS_2_/WSe_2_ PhC nanostructures. Our strategy can manipulate the exciton emission in synthetic energy-momentum domain at the ultimate thickness limit without the PL suppression at the interface (*26*), thereby offering a potential route to explore the interaction of light and the emerging exciton states in the moiré superlattices of vdW semiconductor heterostructures (*39*).

## METHODS

### Sample fabrication

WS_2_ and WSe_2_ 1Ls are first mechanically exfoliated from bulk crystals and then transferred to polydimethylsiloxane stamps. The number of layers of WS_2_ and WSe_2_ are determined by their PL spectra. To prepare twisted WS_2_/WSe_2_ hetero-bilayers, each 1L is characterized by polarization-dependent SHG measurement to determine the crystal orientation and then stacked vertically to form WS_2_/WSe_2_ hetero-bilayers. To fabricate free-standing WS_2_/WSe_2_ PhC nanostructures, we first use focused ion beam milling to fabricate free-standing SiN_x_ PhC nanostructures on a SiN_x_ membrane. After that, we transfer WS_2_/WSe_2_ hetero-bilayers on the SiN_x_ PhC nanostructures. After a series of reactive ion etching and hydrogen fluoride (HF) vapor etching, we can obtain free-standing WS_2_/WSe_2_ PhC nanostructures. More details on the sample fabrication can be found in note S1.

### Optical characterization

All the measurements are conducted in room temperature and atmospheric conditions. To characterize the PL spectra, we optically excite the exciton emission by continuous wave lasers that are focused by an objective with an NA of 0.9 (power: ~200 μW, beam diameter: ~0.90 μm). The PL signal is collected by the same objective and projected on a spectrometer equipped by a cooled charge-coupled device (CCD). For the momentum space characterization, a series of lenses are used to project the Fourier plane of the sample onto the CCD camera. For the polarization-dependent SHG measurement, the samples are pumped by a focused 1.165-eV laser, whose polarization is controlled by a linear polarizer. The SHG signal is collected by an objective with an NA of 0.8 and then filtered by a 2.33-eV narrow band-pass filter. To measure the angle-resolved transmission spectra of PhC nanostructures, an incoherent broadband light source is first partially collimated and then incident on the sample. The sample is rotated while the incident light is fixed to vary the incident angle. The transmitted light is collected by a long working distance objective with an NA of 0.42. More details on the optical characterization can be found in note S10.

## Supplementary Materials

### This PDF file includes:

Supplementary Notes 1 to 10 Figs. S1 to S22 Table S1 References
