# Supplementary material for: Directional sorting of exciton emissions from twisted WS2/WSe2 hetero-bilayers using self-coupled photonic crystal resonances
Source: Sci Adv. 2025 Apr 25;11(17):eadu4968. doi: 10.1126/sciadv.adu4968 (PMC12024640; doi:10.1126/sciadv.adu4968)
Supplement: Supplementary file 1 — Supplementary Notes 1 to 10 Figs. S1 to S22 Table S1 References [file sciadv.adu4968_sm.pdf]

Science Advances
AAAS

Supplementary Materials for

Directional sorting of exciton emissions from twisted WS_2/WSe_2 hetero-bilayers using self-coupled photonic crystal resonances

Yuhua Chen *et al.*

Corresponding author: Xingwang Zhang, xwzhang2021@sinano.ac.cn

Sci. Adv. **11**, eadu4968 (2025)
DOI: 10.1126/sciadv.adu4968

This PDF file includes:

Supplementary Notes 1 to 10
Figs. S1 to S22
Table S1
References

Supplementary Note 1 Device design and fabrication

To design the free-standing photonic crystal (PhC) nanostructured WS_2/WSe_2 hetero-bilayers, we first obtain the complex refractive indices of WS_2/WSe_2 hetero-bilayers, which are described in details in Supplementary Note 4. With the complex refractive indices of WS_2/WSe_2 hetero-bilayers in hand, we then perform numerical simulations using Rigorous Coupled Wave Analysis (RCWA) to calculate the momentum-resolved transmission spectra of the PhC nanostructures. During the simulation, we optimize the lattice constant and hole radius of the PhC nanostructures to make sure that excitons from the twisted WS_2/WSe_2 hetero-bilayers can be well separated in the momentum space.

Figure S1 provides a detailed description of the nanofabrication process of twisted WS_2/WSe_2 PhC nanostructured hetero-bilayers. Firstly, we fabricate PhC nanostructures on a commercially available silicon nitride (SiN_x) membrane by focused ion beam (FIB). The FIB conditions include an acceleration voltage of 30 kV and a beam current of 10 pA. These conditions are optimized to achieve high-resolution patterning of the SiNx PhC nanostructures. Figure S2 shows the scanning electron microscopy (SEM) images of two representative SiN_x PhC membranes. To characterize the optical properties of the suspended SiN_x PhC membranes, we measure the angle-resolved transmission spectra, as shown in Fig. S3. Compared with the simulation results, the PhC resonances are significantly widened, which is attributed to the non-radiative losses, such as surface and edge roughness introduced during the nanofabrication processes.

After fabricating the SiN_x PhC nanostructures, WS_2 and WSe_2 monolayers are mechanically exfoliated from bulk crystals and transferred to polydimethylsiloxane (PDMS) stamps. Then, the WS_2 and WSe_2 monolayers are transferred onto the SiN_x PhC nanostructures to form the WS_2/WSe_2 hetero-bilayers. The transfer process is carried out using a dry-transfer method to ensure good contact between the hetero-bilayers and the PhC nanostructures. During the transfer process, appropriate heating (e.g., at 60 - 80 °C) and application of a certain pressure are carried out to ensure that the monolayers are in full contact with the target substrate. The optical microscopy images for twisted WS_2/WSe_2 bilayers on SiN_x PhC membranes with stacking angles of 30° and 10° are shown in Fig. S4a and S4b.

After that, PhC patterns are transferred to twisted WS_2/WSe_2 bilayers with reactive-ion etching (RIE) by using SiN_x PhC membranes as etch masks. For the RIE etching process, the etching gas is CF_4, and the gas flow rate is maintained at 30 sccm to ensure a stable plasma environment. The etching pressure is kept at 15 mTorr, and the temperature is at 15°C. The RIE power is set at 150 W to achieve a proper etching rate. The total etching time is 18 s, which is carefully controlled to avoid over-etching. Figure S4c and S4d show the optical micrographs of samples after the RIE processes.

Finally, we can remove the SiN_x PhC membranes by using hydrofluoric (HF) vapor etching. The SiN_x membrane serves not only as a mask for the dry etching of twisted WS_2/WSe_2 bilayers, but also as a sacrifice substrate to suspend WS_2/WSe_2 bilayers. We use HF vapor etching for 2 hours at room temperature to remove the remaining SiN_x membrane, leaving only the free-standing WS_2/WSe_2 PhC nanostructures. Figure S4e and S4f show the optical micrographs of samples after HF etching process. Due to the stretching and sinking effects of WS_2/WSe_2 PhC bilayers caused by the suspension, the geometric parameters of PhC nanostructures are further adjusted in accordance with the sinkage of the free-standing bilayers. In this work, the geometric parameters for WS_2/WSe_2 PhC bilayers stacking at 30° and 10° are determined to be Λ = 705 nm, r = 185 nm, and Λ = 700 nm, r = 240 nm, respectively.

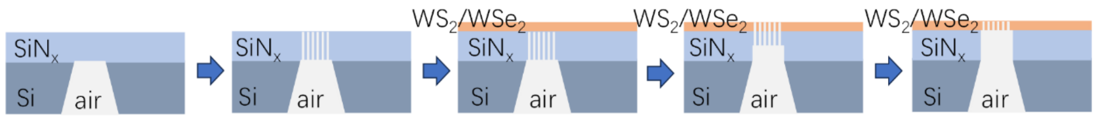

Fig. S1. Fabrication process of twisted WS_2/WSe_2 PhC bilayers.

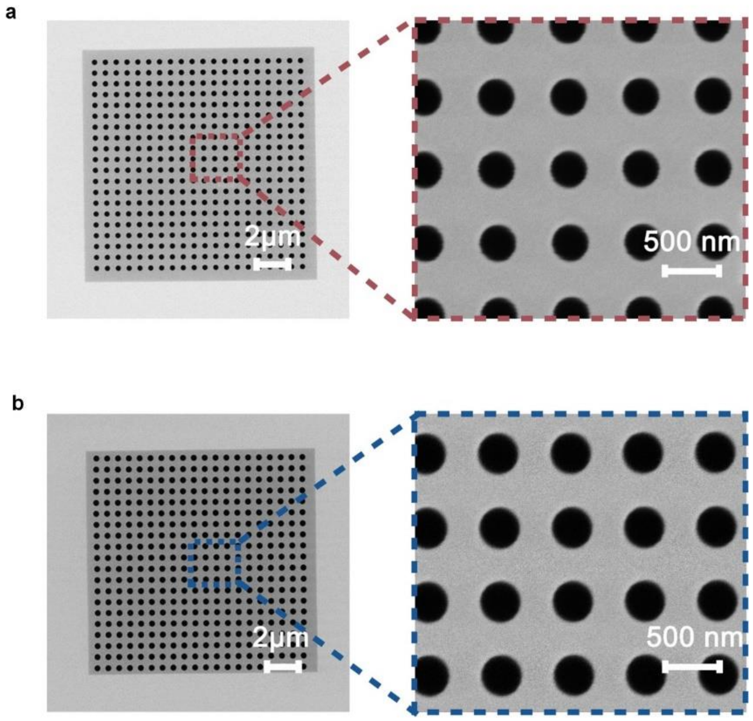

Fig. S2. Scanning electron microscopy (SEM) images of two suspended SiN_x membranes patterned with PhC hole array structures. a, Λ = 676 nm, r = 168 nm. **b,** Λ = 662 nm, r = 225 nm.

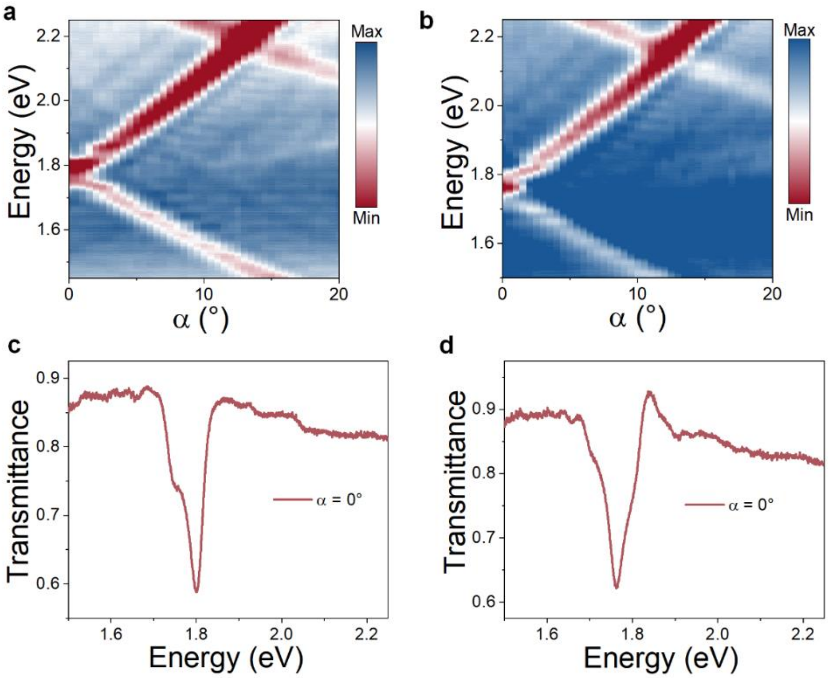

Fig. S3. Transmission spectra of suspended SiN_x PhC membranes. Measured angle-resolved transmission spectra of suspended SiN_x PhC membranes, **a,** θ = 30°, **b,** θ = 10°. Measured transmission spectra of suspended SiN_x PhC membranes under normal incidence, **c,** θ = 30°, **d,** θ = 10°.

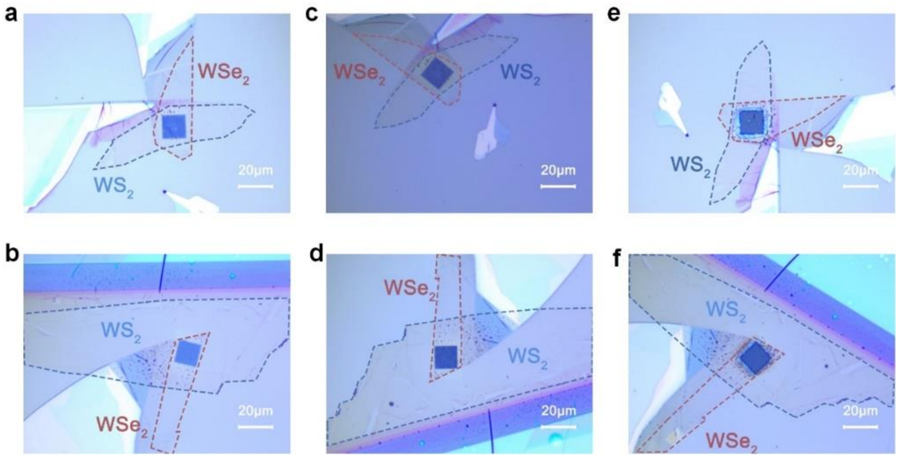

Fig. S4. The optical micrographs of PhC nanostructured WS_2/WSe_2 bilayers. Two twisted WS_2/WSe_2 bilayers stacked at 30° (**a**) and 10° (**b**) on the SiN_x PhC membranes. The optical micrographs of two twisted WS_2/WSe_2 bilayers stacked at 30° (**c**) and 10° (**d**) on the SiN_x PhC membranes after RIE process. The optical micrographs of two suspended WS_2/WSe_2 PhC bilayers stacked at 30° (**e**) and 10° (**f**) after HF etch process. Among them, the area bounded by the red dash line is WSe_2 monolayer while the area bounded by the blue dash line is WS_2 monolayer, and the overlapping area is WS_2/WSe_2 bilayers.

Supplementary Note 2 Polarization dependent second harmonic generation measurement

To control the stacking angle between WS_2 and WSe_2 monolayers, we can first determine the crystal orientations of monolayers by measuring the polarization dependent second harmonic generation (SHG) with a high-power continuous wave 1.165 eV laser. As depicted in Fig. S5a and S5b, the experimental SHG data for WSe_2 and WS_2 are respectively plotted by red and blue dots in polar coordinates, which are further fitted by gray solid lines. During the fitting, the discrepancies between the experimental data and the theoretical six-fold SHG patterns result in fitting errors, which are listed in Table S1. Therefore, the final stacking angles between WS_2 and WSe_2 monolayers have an error < ±1.0°. By this way, we can finally determine the crystal orientations and control the stacking angles between WS_2 and WSe_2 in our experiment.

Since the SHG patterns of both WS_2 and WSe_2 have six-fold rotational symmetry, the case of AA stacking (θ =0°) and AB stacking (θ =60°) cannot be differentiated by separately measuring the SHG of each monolayer. In this case, we can compare the SHG intensity of the hetero-bilayer region with those in the monolayer regions. For the AA (AB) stacking case, the second harmonic field of the two monolayers will constructively (destructively) interfere, giving SHG signal stronger (weaker) than monolayers.(*9*) With this in mind, we prepare twisted WS_2/WSe_2 bilayers with different stacking angles (θ = 10°, 19°, 30°, 41°, 52°). We then measure the SHG in different regions of the sample under the same condition (Fig. S6). Due to the constructive interference of the SHG signals, the SHG of hetero-bilayers is stronger than the constituent monolayers when the stacking angle is smaller than 30° (Fig. S6a and S6b). In contrast, when the stacking angle is between 30° and 60°, the destructive interference of SHG signals results in smaller SHG intensity than the constituent monolayers (Fig. S6c and S6d).

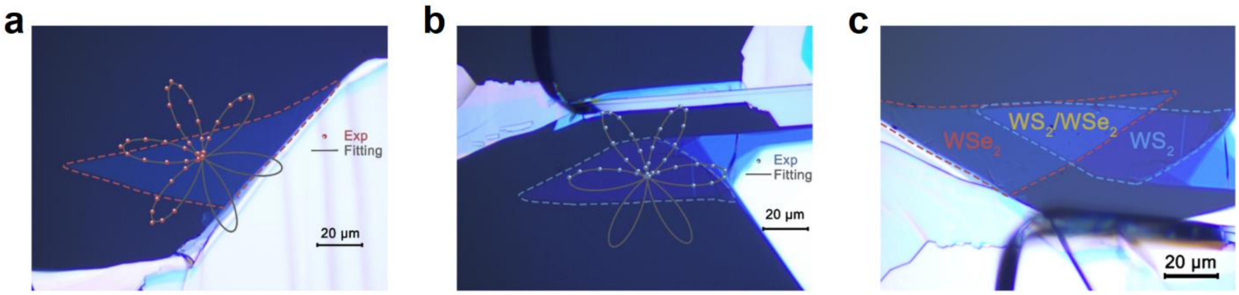

Fig. S5. Polarization dependent second harmonic generation (SHG) measurement. Optical micrographs and polarization dependent SHG patterns with the corresponding fitting curves of WSe_2 (**a**) and WS_2 (**b**) monolayers, respectively. **c,** Optical micrograph for a stacked WS_2/WSe_2 hetero-bilayer.

Table S1 The fitting errors of stacking angles of the hetero-bilayers

1L WS_2	0°±0.26°	0°±0.30°	0°±0.20°	0°±0.43°	0°±0.45°
1L WSe_2	10°±0.32°	19°±0.33°	30°±0.21°	41°±0.28°	52°±0.35°
Twist angle	10°±0.58°	19°±0.63°	30°±0.41°	41°±0.71°	52°±0.80°

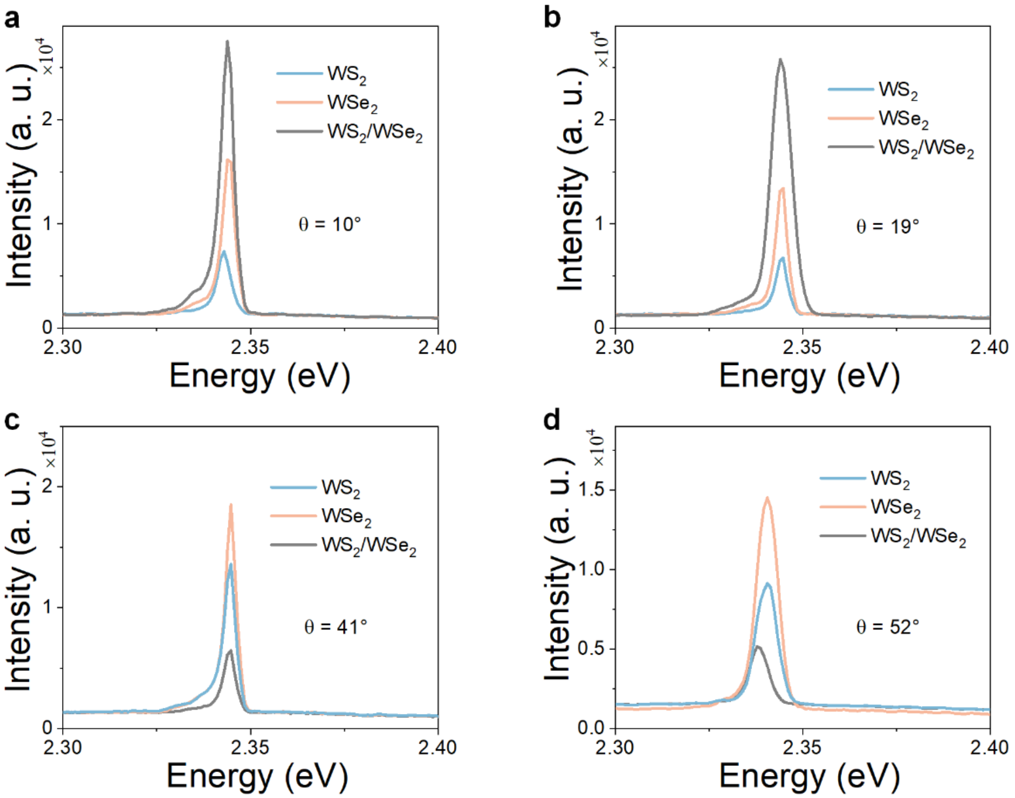

Fig. S6. SHG spectra of different areas (1L WS_2, 1L WSe_2 and WS_2/WSe_2 bilayers) in twisted WS_2/WSe_2 bilayers with different stacking angles. a, θ = 10°. **b,** θ = 19°. **c,** θ = 41°. **d,** θ = 52°.

Supplementary Note 3 Photoluminescence spectra characterization

To rigorously characterize the twist angle dependent photoluminescence (PL) spectra of WS_2/WSe_2 hetero-bilayers, we compare the PL spectra of hetero-bilayer regions with the monolayer regions from the same samples in our experiment (Fig. S7a). As can be seen in Fig. S7b, the PL spectra exhibit a narrow peak centered at 2.02 eV for WS_2 monolayer and a slightly broader peak centered at 1.67 eV for WSe_2 monolayer, both of which originate from direct band gap transitions in monolayers. For the hetero-bilayer region, distinct from the PL spectra from monolayer regions, we observe extra spectral features, which are attributed to the inter-layer interaction between constituent monolayers.

In the meantime, we also plot the PL spectra for twisted WS_2/WSe_2 bilayers, 2L WS_2 and 2L WSe_2 in the same figure for comparison. As shown in Fig. S8a, the PL spectra for WS_2/WSe_2 bilayers exhibit obvious twisted-angle-dependent characteristics. Comparing the PL spectra of WS_2 and WSe_2 monolayers, the PL spectra of twisted WS_2/WSe_2 bilayers display new emission peaks near 1.5 eV, which are attributed to twist-driven excitons (TDE).(*9*, *10*, *29*) More importantly, the WS_2/WSe_2 bilayers with all these stacking angles have much stronger PL intensity than that of bilayer WS_2 and WSe_2 (Fig. S8c). In addition, we note that the PL of hetero-bilayers is usually quenched compared with the monolayer exciton emission due to the ultra-fast inter-layer charge transfer(*30*) and dielectric screening(*31*, *32*). However, compared with the few-layer WS_2 and WSe_2, the hetero-bilayers show much stronger PL intensity (Fig. S8b). Therefore, by stacking 1L WS_2 and WSe_2 together, we can obtain more efficient luminescence and higher tunability than their few-layer counterparts.

In Fig. 2b in the main manuscript, we can observe that there are two more emission peaks near A excitons of 1L WS_2. The one with lower emission energy is attributed to A^- excitons of 1L WS_2.(*40*, *41*) To understand the origin of the one with higher emission energy, we have measured the reflectance spectra of the 1L WS_2 and 1L WSe_2. As can be seen in Fig. S9a, the B exciton resonance of 1L WSe_2 has a slightly higher energy than the A exciton of 1L WS_2. Moreover, we have also measured the PL spectra of 1L WSe_2 in Fig. S9b, and we can observe the B exciton emission of 1L WSe_2 is around 2.15 eV, which is indeed slightly larger than that of A exciton of 1L WS_2 (~2.02 eV). Therefore, we can conclude that the emission peak with a slightly higher energy than A excitons of 1L WS_2 in Fig. 2b in the main manuscript is attributed to the B excitons of 1L WSe_2.

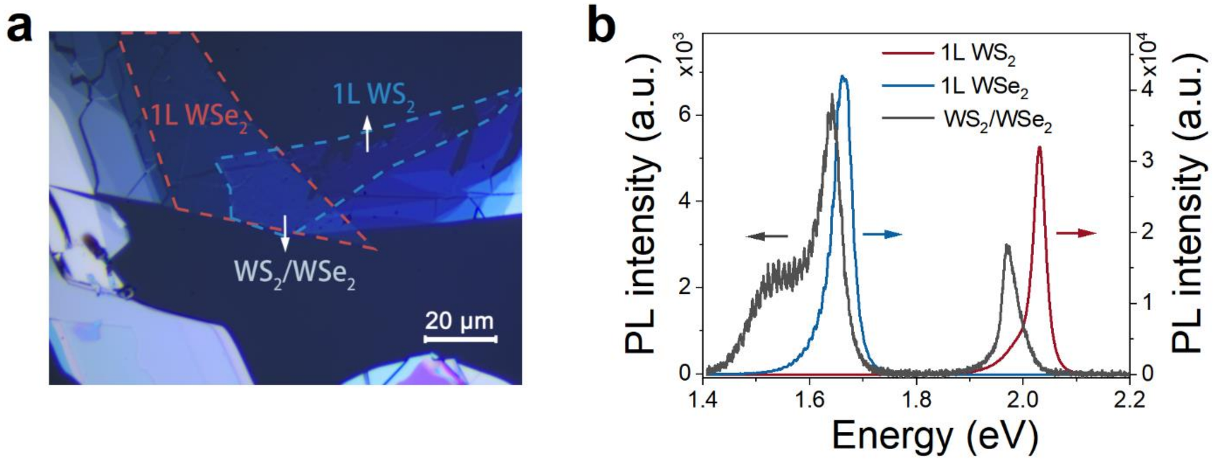

Fig. S7. PL characteristics of WS_2/WSe_2 bilayers, 1L WS_2 and 1L WSe_2 on the same sample. **a,** Optical micrograph of a WS_2 /WSe_2 hetero-bilayer with a stacking angle of 19°; **b,** comparison of PL spectra between WS_2 /WSe_2 hetero-bilayer region and the constituent monolayer regions of the same sample.

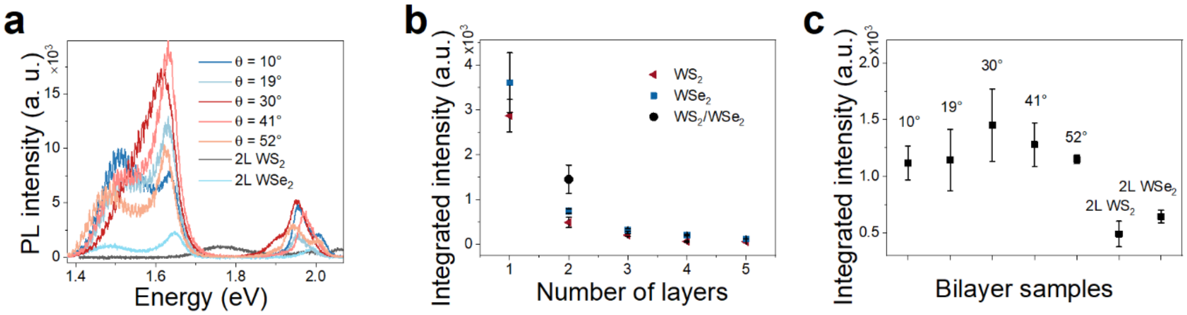

Fig. S8. Photoluminescence spectra characterization of twisted WS_2/WSe_2 bilayers. a, PL spectra comparison of twisted WS_2/WSe_2 bilayers and the mechanically exfoliated bilayers. **b,** Comparison of PL integral intensities for WS_2 of different numbers of layers, WSe_2 of different numbers of layers, and the 30° stacked WS_2/WSe_2 hetero-bilayers. **c,** Comparison of PL integral intensities for WS_2/WSe_2 hetero-bilayers with different stacking angles, WS_2 bilayers and WSe_2 bilayers. The error margins in **b** and **c** are determined by multiple separate measurements.

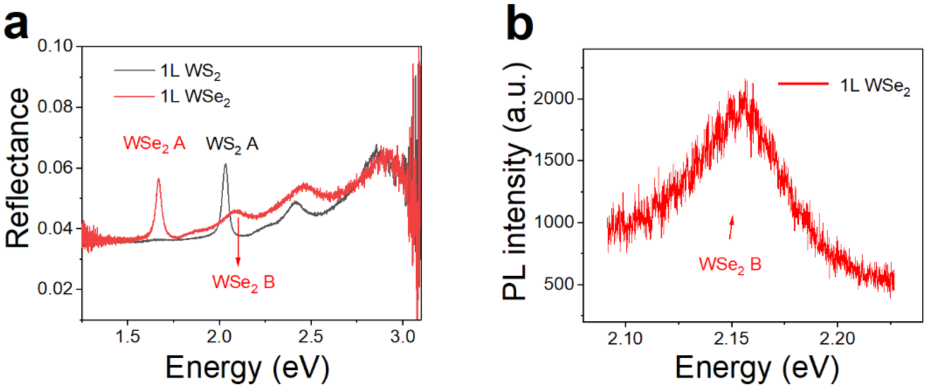

Fig. S9. Photoluminescence spectra characterization of WSe_2 B exciton. a, Reflection spectra for 1L WS_2 and 1L WSe_2. **b,** PL spectra for 1L WSe_2 near the B exciton.

Supplementary Note 4 Dielectric constants measurement for twisted WS_2/WSe_2 hetero-bilayers

Fig. S10a display the measured reflection spectra for WS_2/WSe_2 hetero-bilayers with different stacking angles. Since the energies for WS_2 and WSe_2 excitons have very weak twist-angle dependence, the corresponding reflection peaks for these excitons have no obvious differences at different stacking angles. On the other hand, it is well known that the exciton oscillator strength for TDE is two orders of magnitude smaller than that of the intra-layer excitons due to the reduced transition dipoles(*34–36*). Thus, the reflection spectra have no visible features associated with TDE, even though we can observe TDE emission in the PL spectra.

Then, we can extract the dielectric constants for different stacking angles from their reflection spectra using Kramers–Kronig constrained variational analysis based on the Lorentz model. The dielectric constant of twisted WS_2/WSe_2 hetero-bilayers $\varepsilon(E) = \varepsilon_r(E) + i\varepsilon_i(E)$ can be fitted by multiple resonances using Lorentz model,(*33*)

$$\varepsilon(E) = \varepsilon_B - \sum_{j=1}^{N} \frac{f_j}{E^2 - E_{0j}^2 + iE\Gamma_j} \quad \text{(S1)}$$

where ε_B , f_j , E_{0j} and Γ_j respectively represent the background dielectric constant, oscillator strength, energy and damping constant of the jth oscillator. The refractive index n and the extinction coefficient κ are calculated by taking the square root of the complex dielectric constant

$$\tilde{n} = n + i\kappa = \sqrt{\varepsilon} \quad \text{(S2)}$$

We first set appropriate initial values for all parameters and calculate the reflection spectrum of twisted WS_2/WSe_2 hetero-bilayers by the Transfer-Matrix Method (TMM)(*33*)

$$T = D_0^{-1}\left[\prod_{m=1}^{N} D_m P_m D_m^{-1}\right] D_{N+1} = \begin{bmatrix} T_{11} & T_{12} \\ T_{21} & T_{22} \end{bmatrix} \quad \text{(S3)}$$

the transmission matrix and the propagation matrix can be written as

$$D_{m-1}^{-1} D_m = \begin{bmatrix} 1 & r_{m-1,m} \\ r_{m-1,m} & 1 \end{bmatrix} / t_{m-1,m} \quad \text{(S4)}$$

$$P_m = \begin{bmatrix} \exp(i\delta_m) & 0 \\ 0 & \exp(-i\delta_m) \end{bmatrix} \quad \text{(S5)}$$

where $\delta_m = 2\pi\omega\tilde{n}_m d_m / c$. $r_{m-1,m}$ and $t_{m-1,m}$ are the reflection and transmission coefficients from $(m-1)$th to mth layer, which are calculated according to the Fresnel's law

$$r_{m-1,m} = \frac{\tilde{n}_{m-1} - \tilde{n}_m}{\tilde{n}_{m-1} + \tilde{n}_m} \quad \text{(S6)}$$

$$t_{m-1,m} = \frac{2\tilde{n}_{m-1}}{\tilde{n}_{m-1} + \tilde{n}_m} \quad \text{(S7)}$$

Therefore, the theoretical reflectance R can be calculated by the system transfer matrix elements T_{ij} which can be expressed as a function of the dielectric constant ε and energy E

$$R = \left(\frac{T_{21}}{T_{11}}\right)^2 = R(E, \varepsilon(E)) \quad \text{(S8)}$$

In our experiment, we can measure the reflectance of twisted WS_2/WSe_2 bilayers as a function of energy E

$$R_{exp} = R_{exp}(E) \quad \text{(S9)}$$

At every energy E, the optimized dielectric constant $\varepsilon(E)$ should minimize the mean squared error (MSE)

$$MSE = \sum_i [R_{exp}(E) - R(E, \varepsilon(E))]^2 \quad \text{(S10)}$$

By performing multiple iterations to minimize MSE, the optimal complex dielectric constant of twisted WS_2/WSe_2 bilayers can be extracted. Due to the sample variation, for every WS_2/WSe_2 bilayer used in experiment, the energies of oscillators in Eq. S1 are further adjusted to incorporate the measured spectral position of exciton resonances. As can be seen in Fig. S10b, the dielectric constants for WS_2/WSe_2 hetero-bilayers with different stacking angles exhibit no much differences, which is consistent with the reflection spectra in Fig. S10a.

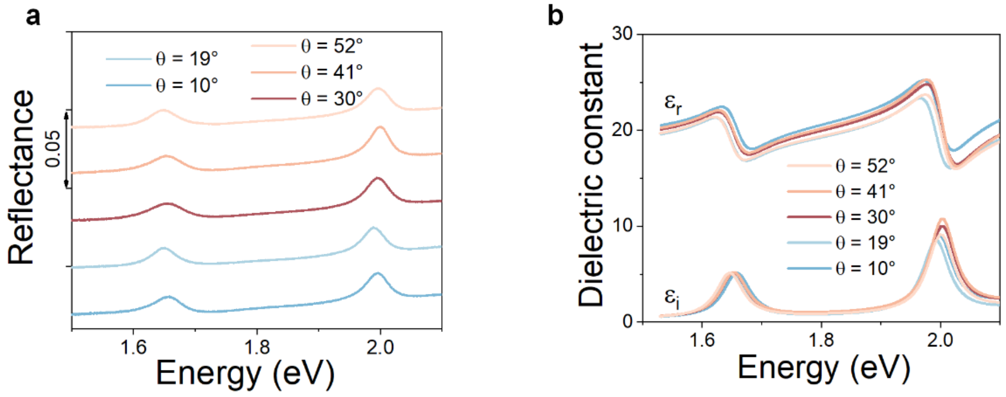

Fig. S10. The measured dielectric constants of twisted WS_2/WSe_2 hetero-bilayers. a, The reflection spectra for WS_2/WSe_2 hetero-bilayers with different stacking angles. The reflection spectra are stacked vertically with a reflectance bias of 0.03. **b,** The extracted dielectric constants for WS_2/WSe_2 hetero-bilayers with different stacking angles from panel **a**.

Supplementary Note 5 Transmission spectra for twisted WS_2/WSe_2 PhC nanostructures

Based on the dielectric constants extracted from the measured reflection spectra of twisted WS_2/WSe_2 bilayers with different stacking angles (θ = 10°, 19°, 30°, 41°, 52°), we can calculate the transmission spectra of twisted WS_2/WSe_2 photonic crystal (PhC) structures (array period Λ = 705 nm and hole radius r = 185 nm) under normal incidence. We notice that the measured guided mode resonances match the simulated spectra very well except for much broader linewidth which is attributed to additional non-radiative losses introduced by the fabrication imperfections (Fig. S11a). In addition, the transmission spectra of WS_2/WSe_2 PhC nanostructures are almost independent upon the stacking angle (Fig. S11b).

We have also evaluated the influence of environment variations on our twisted WS_2/WSe_2 PhC nanostructures, which may introduce changes of geometric parameters and refractive index of the PhC nanostructures.

In our experiment, the twisted WS_2/WSe_2 PhC nanostructures may suffer from thermal fluctuations. We note that the WS_2 and WSe_2 monolayers have thermo-optic coefficients of 1.3×10^{-4} K^{-1} and 2.6×10^{-4} K^{-1}, respectively.(*42*) The thermal expansion coefficients for WS_2 and WSe_2 monolayers are 6.1×10^{-6} K^{-1} and 7.3×10^{-6} K^{-1}, respectively.(*43*) The temperature change in our experiment condition is less than ±5K. We then calculate the transmission spectra of the twisted WS_2/WSe_2 PhC nanostructures after considering the influence of temperature variations in Fig. S12. We can see that the wavelengths of guided mode resonances are only changed by ~±0.025 nm when the temperature is change by ±5K.

The vibrations and fabrication imperfections may result in changes of lattice constant and hole radius in our twisted WS_2/WSe_2 PhC nanostructures. To this end, we calculate the transmission spectra of the twisted WS_2/WSe_2 PhC nanostructures at different lattice constant Λ and hole radius R in Fig. S13. We can see that the wavelengths of guided mode resonances are only changed by ~±0.08 nm when the hole radius changes ±10 nm. However, when the lattice constant is changed by ±10 nm, the wavelengths of guided mode resonances are change by ~±10 nm.

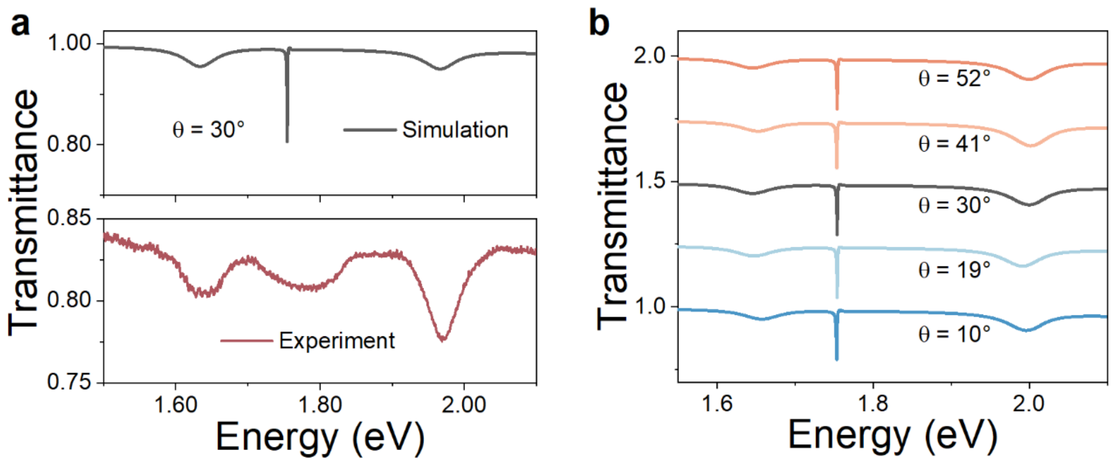

Fig. S11. Transmission spectra for twisted WS_2/WSe_2 PhC nanostructures. a, Simulated and measured transmission spectra for WS_2/WSe_2 PhC nanostructures with a stacking angle of 30°. **b,** Simulated transmission spectra of twisted WS_2/WSe_2 PhC structures (array period Λ = 705 nm and hole radius r = 185 nm) at different stacking angles θ. Each curve is plotted with a transmittance bias of 0.4.

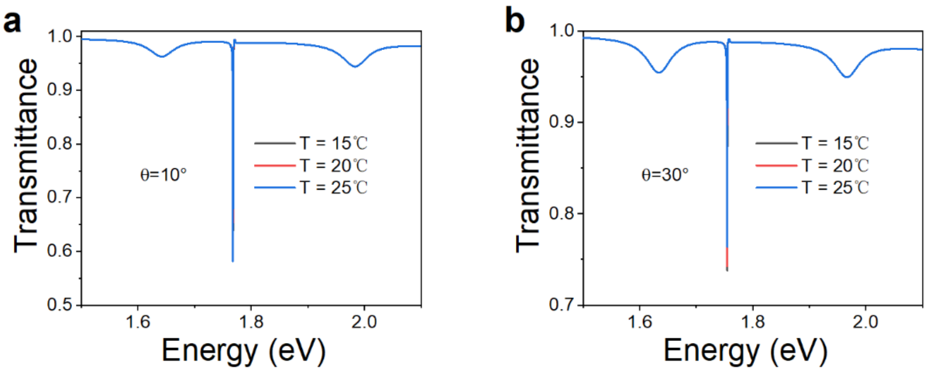

Fig. S12. Simulated transmission spectra of twisted WS_2/WSe_2 PhC structures at different temperatures. a, stacking angle θ=10°. **b,** stacking angle θ=30°.

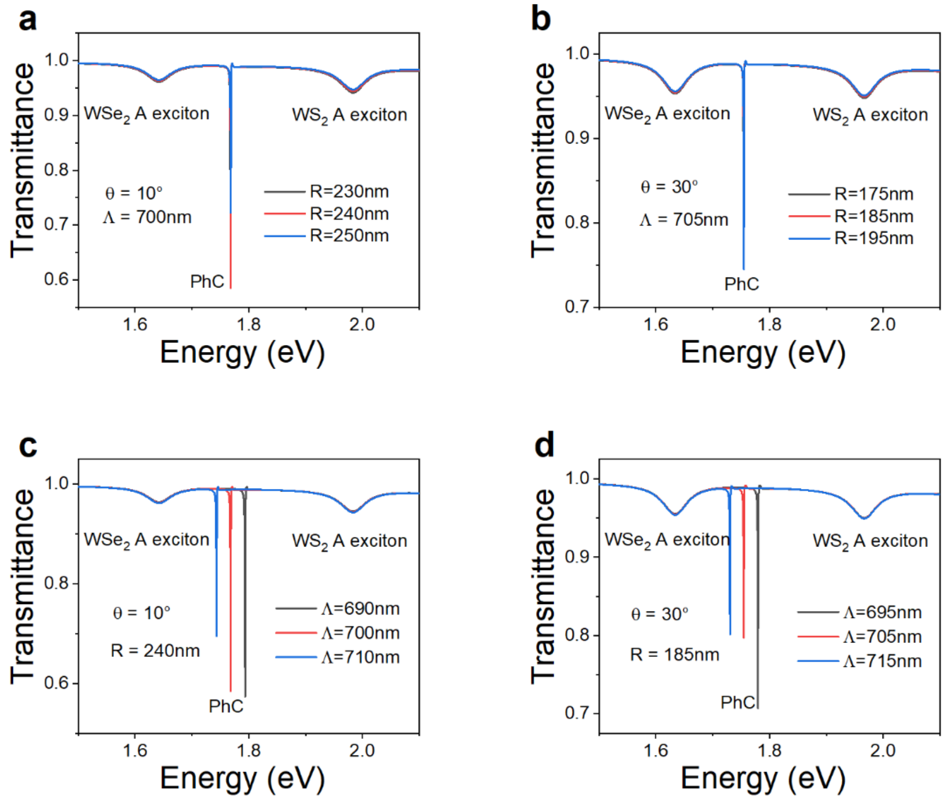

Fig. S13. Simulated transmission spectra of twisted WS_2/WSe_2 PhC structures at different lattice constant Λ and hole radius R. Stacking angle θ=10° (**a, c**). The stacking angle θ=30° (**b, d**).

Supplementary Note 6 Exciton-photon coupling in the free-standing photonic crystal nanostructured WS_2/WSe_2 hetero-bilayers

To elaborate on the exciton-photon coupling in the free-standing photonic crystal (PhC) nanostructured WS_2/WSe_2 hetero-bilayers, we can extract the exciton resonance energy and photon resonance energy from the momentum-resolved transmission spectra of the PhC nanostructured hetero-bilayers, and use the Temporal Coupled-Mode Theory (TCMT) to analyze the dispersion relationship.(*33*) According to the TCMT, the exciton-photon coupling can be described by

$$\begin{pmatrix} \omega_{PhC} + i\gamma_{PhC} & g \\ g & \omega_{Ex} + i\gamma_{Ex} \end{pmatrix} \mathrm{v} = \omega_{\pm}\mathrm{v} \tag{S11}$$

where ω_{PhC} and ω_{Ex} are the resonance frequencies of photons and excitons, respectively. γ_{PhC} and γ_{Ex} denote their damping rates. g is the exciton-photon coupling strength. v describes the Hopfield coefficient, and $\omega_{\pm}$ represent the resonance frequencies of the eigenmodes in the coupling system, which can be derived as

$$\omega_{\pm} = \frac{\omega_{PhC}+\omega_{Ex}}{2} + \frac{i(\gamma_{PhC}+\gamma_{Ex})}{2} \pm \sqrt{g^2 - \frac{1}{4}\left(\gamma_{PhC} - \gamma_{Ex} + i(\omega_{PhC} - \omega_{Ex})\right)^2} \tag{S12}$$

To determine whether the exciton-photon coupling is in the weak or strong coupling regimes, we use the minimum energy separation between the eigenmodes to represent Rabi splitting Ω_R. When $\omega_{PhC} = \omega_{Ex}$, the corresponding Rabi splitting is written as

$$\Omega_R = 2\sqrt{g^2 - \frac{1}{4}(\gamma_{PhC} - \gamma_{Ex})^2} \tag{S13}$$

When the energy exchange rate between excitons and photons in the atomically thin PhC nanostructured WS_2/WSe_2 hetero-bilayers is larger than the damping rates of excitons and photons, the criteria below are satisfied and the exciton-photon coupling is in the strong coupling regime.(*19*, *27*) Otherwise, the exciton-photon coupling is in the weak coupling regime.

$$c_1 = \Omega_R/(\gamma_{PhC} + \gamma_{Ex}) > 1 \tag{S14}$$

$$c_2 = g/\sqrt{(\gamma_{PhC}^2 + \gamma_{Ex}^2)/2} > 1 \tag{S15}$$

Prior to analyze the exciton-photon coupling in the free-standing PhC nanostructured WS_2/WSe_2 hetero-bilayers, we need to obtain the momentum-resolved transmission spectra for photons in bare PhC nanostructures without exciton contributions. With this in mind, we have artificially removed the contributions of WS_2 A exciton and WSe_2 A exciton in the dielectric constants of the hetero-bilayers (Fig. S14).(*19*, *27*, *33*) With the dielectric constants shown in Fig. S14, we can calculate the momentum-resolved transmission spectra for PhC nanostructures without and with exciton contributions using RCWA. After that, we can extract the momentum dispersion relationships for WS_2 A exciton, WSe_2 A exciton, bare PhC nanostructures without exciton contributions from the simulated momentum-resolved transmission spectra, as shown in Fig. S15. Meanwhile, the linewidths of the PhC and exciton resonances are obtained from simulations as γ_{PhC}=7 meV, γ_{Ex-WS2}=50 meV and $\gamma_{Ex-WSe2}$=55 meV, respectively. Then, we utilize Eq. S12 to calculate the dispersion of eigenmodes in the coupling system. By adjusting the coupling strength g, we can fit the dispersion of eigenmodes with the calculated momentum-resolved transmission spectra of PhC nanostructures with exciton contributions. According to the TCMT fitting, the Rabi splitting values are calculated as 12 meV in Fig. S15a, 6 meV in Fig. S15b, 11 meV in Fig. S15c, 6 meV in Fig. S15d, respectively. Considering the criteria in Eq. S14 and S15, we can conclude that the exciton-photon coupling in the free-standing PhC nanostructured WS_2/WSe_2 hetero-bilayers is in the weak coupling regime.

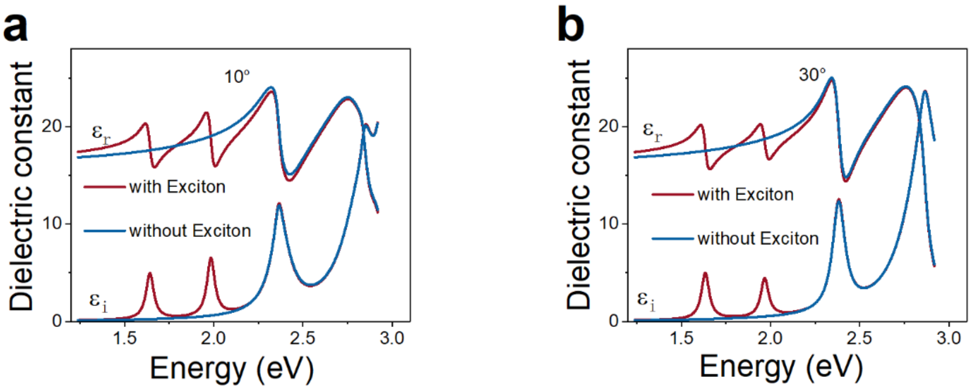

Fig. S14. Dielectric constants for WS_2/WSe_2 hetero-bilayers. a, stacking angle θ=10°. **b,** stacking angle θ=30°.

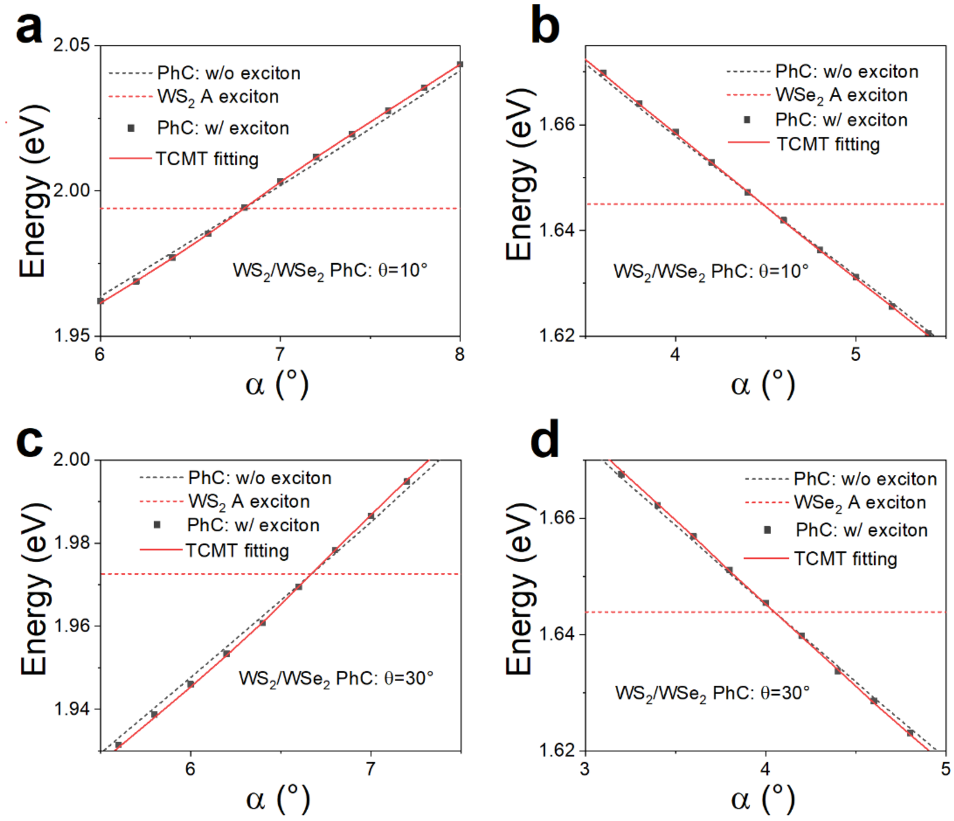

Fig. S15. Momentum dispersion relationships for free-standing PhC nanostructured WS_2/WSe_2 hetero-bilayers. Stacking angle θ=10° **(a-b)** and θ=30° **(c-d)**.

We note that there is usually Purcell effect enhancement in the weak coupling regime for resonant photonic nanostructures. Bearing this in mind, to further analyze the exciton-photon coupling in the free-standing PhC nanostructured WS_2/WSe_2 hetero-bilayers in the weak coupling regime, we calculate the Purcell factors in the free-standing PhC nanostructured WS_2/WSe_2 hetero-bilayers by using FDTD, and compare them with those in unpatterned free-standing WS_2/WSe_2 hetero-bilayers.(*21*) As can be seen in Fig. S16, due to the resonant enhancement of the PhC nanostructures, the Purcell factors are respectively enhanced by 1.81, 1.90, and 2.47 times for the WS_2 A exciton, WSe_2 A exciton and TDE in the PhC nanostructured WS_2/WSe_2 hetero-bilayers with a stacking angle of 10°. Similarly, for the PhC nanostructured WS_2/WSe_2 hetero-bilayers with a stacking angle of 30°, the Purcell factor enhancements are respectively 2.08, 1.60, and 2.28 for the WS_2 A exciton, WSe_2 A exciton and TDE. Therefore, although the PhC nanostructures are

atomically thin, the coupling between guided mode resonances and excitons can still achieve relatively high Purcell factor enhancement.

On the other hand, as the guided mode resonance is above the light line, it can efficiently couple photons from the near field to the far field. To this end, we have also calculated the extraction rates in the free-standing PhC nanostructured WS_2/WSe_2 hetero-bilayers by using FDTD, and compare them with those in unpatterned free-standing WS_2/WSe_2 hetero-bilayers. Such extraction rates are utilized to evaluate how much exciton emission can be extracted from the nanostructures to the far field.(*21*) As can be seen in Fig. S16, in the presence of PhC nanostructures, the extraction rates are respectively enhanced by 4.82, 4.07, and 2.91 times for the WS_2 A exciton, WSe_2 A exciton and TDE in the hetero-bilayers with a stacking angle of 10°. Similarly, for the PhC nanostructured WS_2/WSe_2 hetero-bilayers with a stacking angle of 30°, the extraction rate enhancements are respectively 2.98, 3.04, and 2.41 for the WS_2 A exciton, WSe_2 A exciton and TDE. Therefore, due to the far field coupling nature of guided mode resonances, the free-standing PhC nanostructured WS_2/WSe_2 hetero-bilayers can efficiently extract the near field excited exciton emission to the far field.

As a summary, in our free-standing PhC nanostructured WS_2/WSe_2 hetero-bilayers, the exciton-photon coupling is in the weak coupling regime. The presence of guided mode resonances in the free-standing WS_2/WSe_2 hetero-bilayers can not only enhance the Purcell factors but also extraction rates of excitons, which can resonantly enhance the exciton emission at the atomic thickness scale.

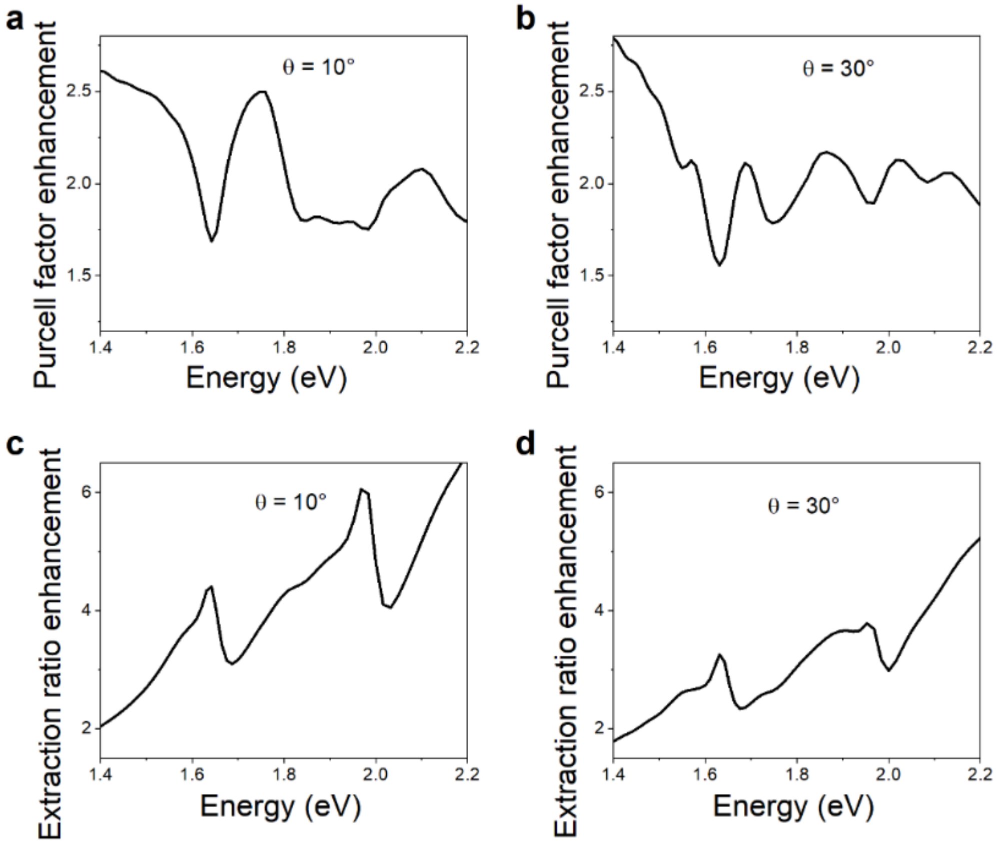

Fig. S16. Exciton emission enhancement analysis in free-standing PhC nanostructured WS_2/WSe_2 hetero-bilayers. The Purcell factor enhancement for free-standing PhC nanostructured WS_2/WSe_2 hetero-bilayers with a stacking angle of θ=10° **(a)** and θ=30° **(b)**. The extraction ratio enhancement for free-standing PhC nanostructured WS_2/WSe_2 hetero-bilayers with a stacking angle of θ=10° **(c)** and θ=30° **(d)**.

Supplementary Note 7 Momentum dispersion of atomically thin PhC nanostructures

The presence of the air hole array patterned in atomically thin PhC nanostructures creates a spatially periodic modulation of the dielectric constant, which introduces scattering loss and folds the waveguide mode dispersion back to the first Brillouin zone, leading to the generation of guided mode resonances above the light line. Consider the light guiding along the *x* axis of an infinitely large slab waveguide placed in the *xy*-plane. The dielectric constants of upper and lower claddings are ε_1 and ε_3, and the dielectric constant of PhC nanostructures, ε_2, is periodically modulated as

$$\varepsilon_2(x+\Lambda) = \varepsilon_L + (\varepsilon_H - \varepsilon_L) f(x) \qquad \text{(S16)}$$

where $f(x)$ is 0 and 1, depending on whether the dielectric constant is ε_H or ε_L for a particular value of *x*.(*24*)

For TE polarization, the total electric field inside the slab can be expressed as a Fourier expansion of harmonic fields propagating along the *x*-axis,(*24*)

$$E_{y,2}(x,z) = \sum_{m=-\infty}^{+\infty} \hat{S}_m(z) e^{-jk_{x,2}x} \qquad \text{(S17)}$$

$$\hat{S}_m(z) = \sum_{p=-\infty}^{+\infty} C_{p,m} e^{-j\xi_p z} \qquad \text{(S18)}$$

where $\hat{S}_{m+h}(z)$ is the amplitude of the inhomogeneous plane wave of the mth space harmonic. p is the transverse mode order. $k_{x,2}$ is determined by the Floquet condition $k_{x,2} = k_0(\sqrt{\varepsilon_g}\sin\theta_2 + \frac{m\lambda}{\Lambda})$, in which ε_g is the average dielectric constant of the PhC nanostructures, and θ_2 is refraction angle inside the slab through the Snell's law. According to Eq. S17, we can see that the electric field inside the PhC nanostructures is modulated by the periodic structure, and generates a series of harmonic components. The coupled-wave equations governing the wave propagation in the PhC nanostructures can be written as

$$\frac{d^2\hat{S}_m(z)}{dz^2} + (k_0^2\varepsilon_g - k_{x,2}^2)\hat{S}_m(z) + jk_0^2(\varepsilon_H - \varepsilon_L)\sum_{h=1,3,5\cdots}^{+\infty}\frac{1}{\pi h}\left[\hat{S}_{m+h}(z) - \hat{S}_{m-h}(z)\right] = 0, \qquad \text{(S19)}$$

For atomically thin PhC nanostructures, the dielectric constants are weakly modulated, i.e. $\varepsilon_H - \varepsilon_L \to 0$, Eq. S19 can be written as

$$\frac{d^2E_{y,2}(z)}{dz^2} + \left(k_0^2\varepsilon_g - \beta^2\right)E_{y,2}(z) = 0, \qquad \text{(S20)}$$

$$\beta = k_{x,2} = k_0(\sqrt{\varepsilon_g}\sin\theta_2 + \frac{m\lambda}{\Lambda}), \qquad \text{(S21)}$$

where β is the propagation constant. When $\varepsilon_1 = \varepsilon_3$, considering the Snell's law, $\sqrt{\varepsilon_1}sin\theta_1 = \sqrt{\varepsilon_g}sin\theta_2$, the momentum matching condition for coupling incident light at an angle θ_1 into a guided mode resonance of the atomically thin PhC nanostructures can be expressed as

$$k_0\sqrt{\varepsilon_1} = k_0(\sqrt{\varepsilon_1}\sin\theta_1 + \frac{m\lambda}{\Lambda}), \qquad \text{(S22)}$$

The mode index of the guided mode resonance is a complex number, $N_{eff} = n_{eff} + j\kappa_{eff}$, where the diffraction and absorption losses contribute to κ_{eff}, and the real part of mode index can be expressed as

$$n_{eff} = k_0\left(\sqrt{\varepsilon_1}\sin\theta_1 + \frac{m\lambda}{\Lambda}\right), m = 0, \pm1, \pm2, ..., \qquad \text{(S23)}$$

In the case of light guiding in the *xy*-plane of a free-standing atomically thin PhC nanostructured, the momentum matching condition becomes,

$$\beta_x = k_x + m\frac{2\pi}{\Lambda}, m = 0,1,2 ..., \qquad \text{(S24)}$$

$$\beta_y = k_y + n\frac{2\pi}{\Lambda}, n = 0,1,2 ..., \qquad \text{(S25)}$$

$$\beta = k_0 n_{eff} \qquad \text{(S26)}$$

$$|\beta|^2 = \left|\beta_x\vec{x} + \beta_y\vec{y}\right|^2 \qquad \text{(S27)}$$

where β_x and β_y are the *x* and *y* components of the propagation constant β of the guided mode resonance. Then, we can derive the momentum dispersion of atomically thin PhC nanostructures,

$$n_{eff}^2 = \left(\frac{k_x}{k_0} + m\frac{2\pi}{\Lambda k_0}\right)^2 + \left(\frac{k_y}{k_0} + n\frac{2\pi}{\Lambda k_0}\right)^2 \qquad \text{(S28)}$$

Clearly, the equi-frequency contours of the guided mode resonances are arcs centered at $(-m\frac{2\pi}{\Lambda k_0}, -n\frac{2\pi}{\Lambda k_0})$ with a radius of n_{eff} in momentum space.

To further elaborate on the physical mechanisms on the manipulation of exciton emission by the self-coupled guided mode resonances in atomically thin PhC nanostructures, we have also simulated how excitons couple to guided mode resonances in the near field and how the guided mode resonances direct exciton emissions to the far field by using FDTD. To this end, we place the free-standing PhC nanostructured WS_2/WSe_2 hetero-bilayer with a stacking angle of 10° in the z = 0 plane, and set an electric dipole at y = -6 μm inside the hetero-bilayer to mimic the exciton. As can be seen in Fig. S17a-c, we can observe that light emitted by the exciton is guided along the atomically thin PhC nanostructures with partial leakage, indicating the far field coupling of guided mode resonances. Such a far field coupling ability of guided mode resonances is due to the fact that the presence of PhC nanostructures introduces an extra momentum, enabling the momentum matching condition between the wave vector inside the hetero-bilayer and the free-space wave vector (Eq. S22). In contrast, for the unpatterned free-standing WS_2/WSe_2 hetero-bilayer, due to the momentum mismatch, light emitted from the exciton is guided along the hetero-bilayer without any leakage (Fig. S17d-f).

To understand the directional sorting of exciton emissions in the momentum space, we record the electric and magnetic fields of a plane that is 5 μm above the hetero-bilayer and 6 μm away from the exciton source to calculate the far field projection. As predicted by Eq. S28, we can observe that the light emitted from the free-standing PhC nanostructured WS_2/WSe_2 hetero-bilayer exbibits as an arc, whose position in the momentum space is energy-dependent (Fig. S18). For the WS_2 A exciton, WSe_2 A exciton and TDE in the free-standing PhC nanostructured WS_2/WSe_2 hetero-bilayer with a stacking angle of 10°, we note that their peak energies are 2.01 eV, 1.62 eV, and 1.52 eV, respectively. As indicated in Fig. S18, the top of the arcs (i.e. $k_x/k_0 = 0$) locate at $k_y/k_0 = 0.113$, -0.083, and -0.153 in the momentum space accordingly, which agree well with the results in Fig. 4b that calculated by Eq. S28. In addition to this, as the field monitor plane is 5 μm above the hetero-bilayer, the light directly emitted from the exciton source can also be collected by the field monitor with a large angle by free space radiation, leading to bright regions at the top side of the Fourier plane.

In summary, light emitted from excitons in the free-standing WS_2/WSe_2 hetero-bilayers can couple to the guided mode resonances via near field interaction. The presence of PhC nanostructures in the free-standing WS_2/WSe_2 hetero-bilayers introduces an extra in-plane momentum, which enables momentum matching condition between the wave vector inside the hetero-bilayer and the free-space wave vector. Leveraging the unique momentum dispersion property of the atomically thin PhC nanostructures, light emitted from excitons is coupled to the free-space with an energy-dependent position in the momentum space, which is determined by the momentum matching condition. Consequently, both intra-layer and inter-layer driven exciton emissions are resonantly separated in the energy-momentum space.

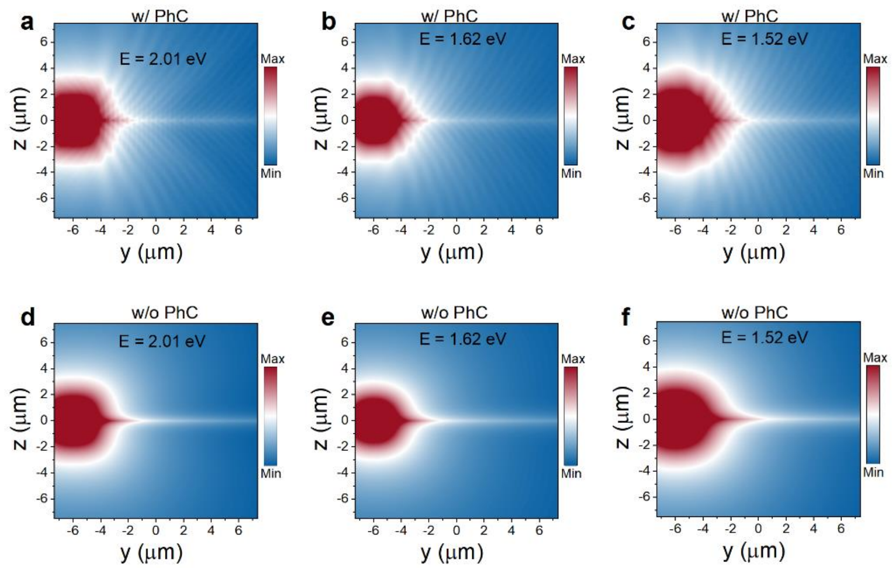

Fig. S17. The calculated electric field distributions for the free-standing WS_2/WSe_2 hetero-bilayer with a stacking angle of θ=10° with (a-c) and without (d-f) PhC nanostructures. The emission peak energies of the WS_2 A exciton, WSe_2 A exciton and TDE are 2.01 eV, 1.62 eV, and 1.52 eV, respectively. The hetero-bilayer is placed at the *z* = 0 plane, and an electric dipole is set at *y* = -6 μm inside the hetero-bilayer to mimic the exciton source.

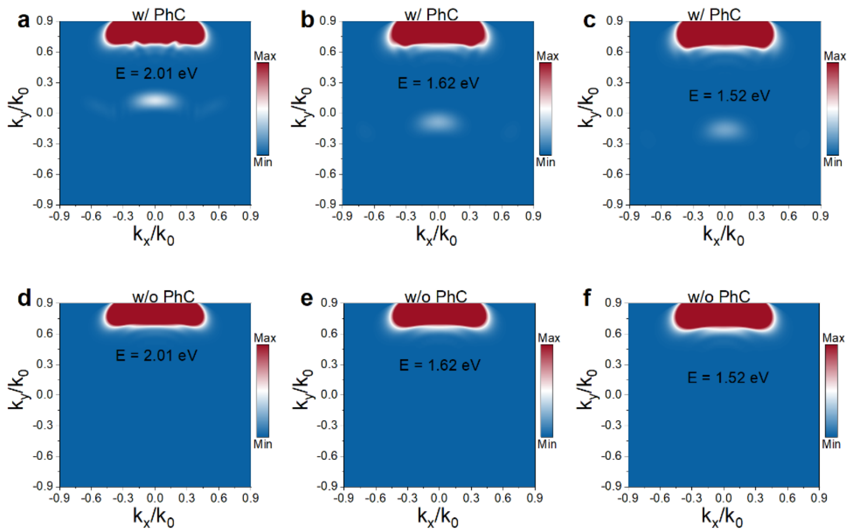

Fig. S18. The calculated exciton emission from the free-standing WS_2/WSe_2 hetero-bilayer with a stacking angle of θ=10° with (a-c) and without (d-f) PhC nanostructures in the momentum space. The emission peak energies of the WS_2 A exciton, WSe_2 A exciton and TDE are 2.01 eV, 1.62 eV, and 1.52 eV, respectively. The hetero-bilayer is placed at the *z* = 0 plane, and an electric dipole is set at *y* = -6 μm inside the hetero-bilayer to mimic the exciton source. We record the electric and magnetic fields of a plane that is 5 μm above the hetero-bilayer and 6 μm away from the exciton source to calculate the far field projection.

Supplementary Note 8 Selective exciton excitation in twisted WS_2/WSe_2 PhC nanostructures

Since excitons differ from each other in the resonant energies, we can selectively excite exciton emissions by using appropriate laser wavelength. With this in mind, we first change the excitation laser energy from 2.33 eV to 1.88 eV. Such an excitation energy is below the WS_2 A exciton but above the WSe_2 A exciton and TDE exciton. Therefore, by using 1.88 eV excitation, we can selectively eliminate the WS_2 A exciton emission. As a result, for the WS_2/WSe_2 hetero-bilayer with a stacking angle of 30°, whose TDE emission energy is quite close to that of WSe_2 A exciton, only one emission spot around 1.62 eV can be observed in Fig. S19a. Similarly, for the WS_2/WSe_2 hetero-bilayers with a stacking angle of 10°, the TDE emission energy is well separated with that of WSe_2 A exciton. Therefore, both the WSe_2 A exciton and TDE emissions can be observed in Fig. S19b. By further reducing the excitation laser energy, it is possible to solely excite and isolate the TDE emission. To this end, we change the excitation laser energy to 1.58 eV, which is below those of WS_2 A exciton (2.01 eV) and WSe_2 A exciton (1.62 eV). Therefore, only TDE emission can be excited in the WS_2/WSe_2 hetero-bilayers. For this reason, for the WS_2/WSe_2 hetero-bilayer with a stacking angle of 30°, whose TDE emission energy (1.61 eV) is above that of excitation laser, we cannot observe any PL signal in the energy-momentum domain. In contrast, when the stacking angle is 10°, the TDE emission energy (1.52 eV) is below that of the excitation laser, so we can observe that only the TDE emission is excited in Fig. S19c.

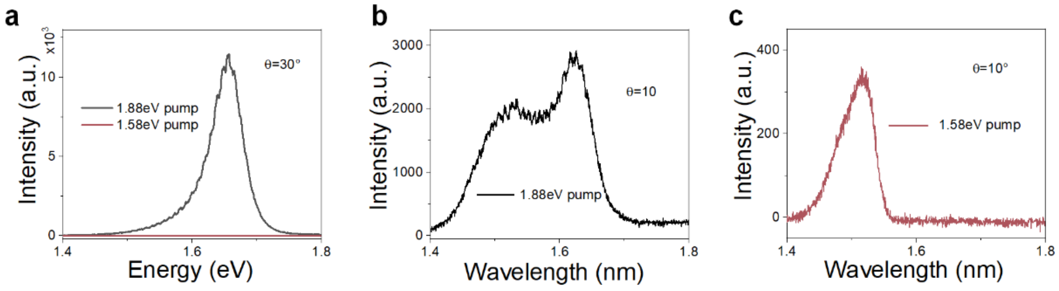

Fig. S19. Exciton emissions in twisted WS_2/WSe_2 PhC nanostructures under different pump energies. a, PL spectra of the WS_2/WSe_2 PhC nanostructures with a stacking angle of 30° under 1.88 eV (gray solid line) and 1.58 eV (red solid line) laser excitations. PL spectra of the WS_2/WSe_2 PhC nanostructures with a stacking angle of 10° under 1.88 eV (**b**) and 1.58 eV (**c**) laser excitations.

Supplementary Note 9   Comparison with other exciton emission control methods

Currently, there are mainly two kinds of methods to control exciton emission from 2D semiconductors. First, by integrating 2D semiconductors onto conventional 3D material photonic nanostructures to construct 2D/3D integrated photonic nanostructures, the optical modes of conventional 3D material photonic nanostructures can interact with excitons in the integrated 2D semiconductors and control the exciton emission properties.(*17*, *20–23*) However, the surfaces of conventional 3D materials are not atomically flat, and there are usually many dangling bonds on the surfaces, which can introduce defect-assisted nonradiative exciton recombination and suppress the exciton emission intensity. Second, by integrating narrow bandgap 2D semiconductors onto photonic nanostructures patterned in broad bandgap 2D semiconductor film to construct all van der Waals integrated photonic nanostructures, the optical modes of photonic nanostructures can interact with excitons in the integrated narrow bandgap 2D semiconductors and control the exciton emission properties.(*44*, *45*) Although the surface of broad bandgap 2D semiconductor film is atomically flat and free from dangling bonds, the high dielectric constant of broad bandgap 2D semiconductor film causes strong dielectric screening effects that significantly reduce the binding energies of excitons in the integrated narrow bandgap 2D semiconductors.

Compared with the above exciton emission control methods, our method can effectively solve the drawbacks mentioned above. First, in contrast to integrating 2D semiconductors with external photonic nanostructures, our WS_2/WSe_2 PhC nanostructures are suspended in the air, which essentially eliminates the potential luminescence suppression effects suffered from the contact interfaces. In addition to this, the environment dielectric constant in our case is reduced to 1.0, which can significantly reduce the dielectric screening effects, leading to remarkably enhanced binding energies of excitons. Second, the free-standing PhC nanostructured WS_2/WSe_2 hetero-bilayers not only provide collective exciton emissions, but also offer photonic resonances that can self-couple to the excitons to manipulate exciton emissions at the atomic thickness scale. Third, leveraging the unique momentum dispersion of the free-standing PhC nanostructured WS_2/WSe_2 hetero-bilayers, both intra-layer and inter-layer driven luminescence are resonantly separated in the energy-momentum space.

Supplementary Note 10 Optical characterization

Our PL experimental setup is schematically shown in Fig. S20. A continuum wave (CW) solid state laser (2.33 eV) is focused on the sample by a 100X objective (NA=0.90), and the exciton emission is filtered by a long-pass filter (F1). A two-dimensional slit is used to select the testing area on the sample. A series lenses (L1, L2, L3 and TL) are used to project the image plane or the Fourier plane onto the CCD of the spectrometer.

To measure the angle-resolved transmission spectra, a white a light source (White Light) is partially collimated and illuminated on the sample after passing through a broad band linear polarizer. The sample is placed on a rotatable stage to adjust the incident angle. The transmitted light is collected by a long working distance objective (50X, NA=0.42). After passing through another broad band linear polarizer, the transmitted light is projected onto a spectrometer equipped with an electron-multiplying CCD with the help of a series of lenses (L1, L2 and TL) (Fig. S21).

To measure the polarization-resolved SHG, a high-power CW laser (1.165 eV) is used as the pump laser (Fig. S22). A linear polarizer is placed on a rotational stage to adjust the polarization of the pump laser. Then, the linearly polarized laser is focused on the sample by a 20X objective (NA=0.45). The SHG signal and the residual pump signal are collected by a 50X objective (NA=0.80). A 2.33 eV band-pass filter is used to extract the SHG signal to increase signal-to-noise ratio. Then, the required polarized SHG signal is extracted through another linear polarizer. A two-dimensional slit is used to select the testing area on the sample. A series image lenses (L1, L2 and TL) are used to project the image plane onto the CCD of the spectrometer.

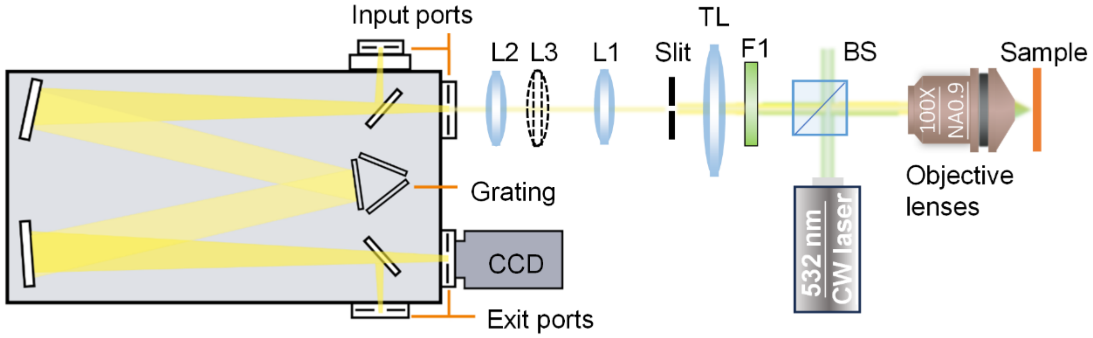

Fig. S20. Experimental setup for PL measurement. L1-L3: Lens. F1: Filter. TL: Tube Lens. BS: Beam Splitter. CCD: Electron-multiplying CCD.

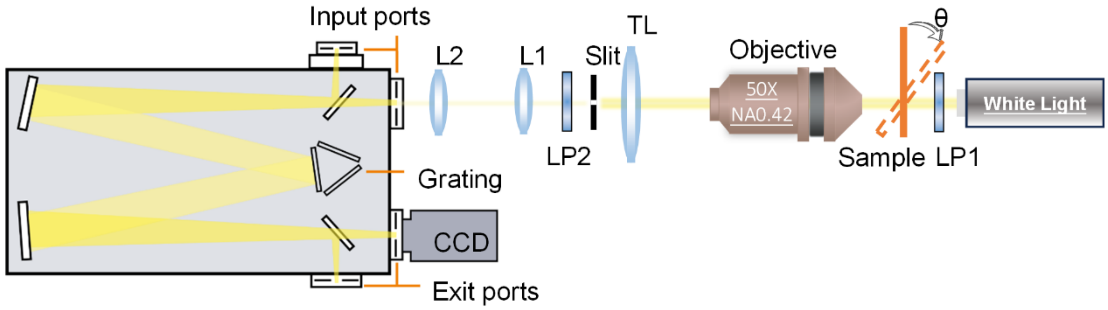

Fig. S21. Experimental setup for transmission spectra measurement. L1 and L2: Lens. LP1 and LP2: Linear Polarizer. TL: Tube Lens. CCD: Electron-multiplying CCD.

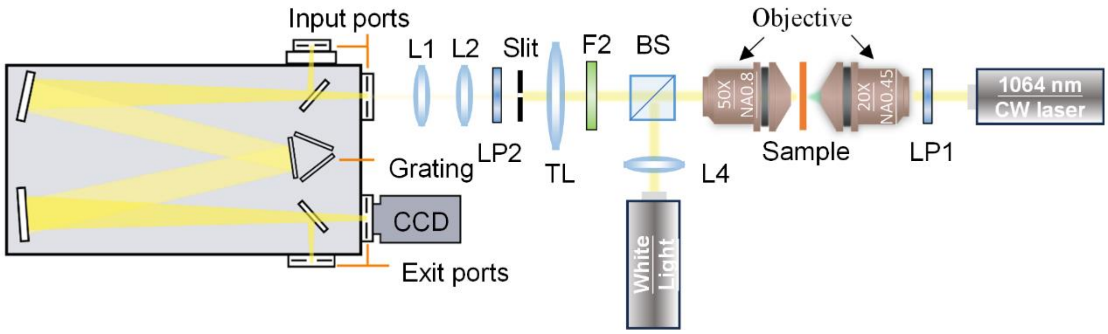

Fig. S22. Experimental setup for SHG measurement. L1, L2 and L4: Lens. LP1 and LP2: Linear Polarizer. TL: Tube Lens. F2: Filter. BS: Beam Splitter. CCD: Electron-multiplying CCD.

REFERENCES AND NOTES

1. Y. Liu, N. O. Weiss, X. Duan, H.-C. Cheng, Y. Huang, X. Duan, Van der Waals heterostructures and devices. *Nat. Rev. Mater.* **1**, 16042 (2016).

2. J. F. Sierra, J. Fabian, R. K. Kawakami, S. Roche, S. O. Valenzuela, Van der Waals heterostructures for spintronics and opto-spintronics. *Nat. Nanotechnol.* **16**, 856–868 (2021).

3. J. Zhang, L. Du, S. Feng, R.-W. Zhang, B. Cao, C. Zou, Y. Chen, M. Liao, B. Zhang, S. A. Yang, G. Zhang, T. Yu, Enhancing and controlling valley magnetic response in MoS_2/WS_2 heterostructures by all-optical route. *Nat. Commun.* **10**, 4266 (2019).

4. S. Wang, X. Cui, C. Jian, H. Cheng, M. Niu, J. Yu, J. Yan, W. Huang, Stacking-engineered heterostructures in transition metal dichalcogenides. *Adv. Mater.* **33**, 2005735 (2021).

5. E. C. Regan, D. Wang, E. Y. Paik, Y. Zeng, L. Zhang, J. Zhu, A. H. MacDonald, H. Deng, F. Wang, Emerging exciton physics in transition metal dichalcogenide heterobilayers. *Nat. Rev. Mater.* **7**, 778–795 (2022).

6. P. Rivera, H. Yu, K. L. Seyler, N. P. Wilson, W. Yao, X. Xu, Interlayer valley excitons in heterobilayers of transition metal dichalcogenides. *Nat. Nanotechnol.* **13**, 1004–1015 (2018).

7. C. Jin, E. C. Regan, D. Wang, M. I. B. Utama, C.-S. Yang, J. Cain, Y. Qin, Y. Shen, Z. Zheng, K. Watanabe, T. Taniguchi, S. Tongay, A. Zettl, F. Wang, Identification of spin, valley and moiré quasi-angular momentum of interlayer excitons. *Nat. Phys.* **15**, 1140–1144 (2019).

8. Y. Liu, A. Elbanna, W. Gao, J. Pan, Z. Shen, J. Teng, Interlayer excitons in transition metal dichalcogenide semiconductors for 2D optoelectronics. *Adv. Mater.* **34**, 2107138 (2022).

9. C. Jin, E. C. Regan, A. Yan, M. I. B. Utama, D. Wang, S. Zhao, Y. Qin, S. Yang, Z. Zheng, S. Shi, K. Watanabe, T. Taniguchi, S. Tongay, A. Zettl, F. Wang, Observation of moiré excitons in WSe_2/WS_2 heterostructure superlattices. *Nature* **567**, 76–80 (2019).

10. K. Wu, H. Zhong, Q. Guo, J. Tang, J. Zhang, L. Qian, Z. Shi, C. Zhang, S. Yuan, S. Zhang, H. Xu, Identification of twist-angle-dependent excitons in WS_2/WSe_2 heterobilayers. *Natl. Sci. Rev.* **9**, nwab135 (2022).

11. S.-H. Cao, W.-P. Cai, Q. Liu, Y.-Q. Li, Surface plasmon-coupled emission: What can directional fluorescence bring to the analytical sciences? *Annu. Rev. Anal. Chem.* **5**, 317–336 (2012).

12. Y.-Y. Xie, P.-N. Ni, Q.-H. Wang, Q. Kan, G. Briere, P.-P. Chen, Z.-Z. Zhao, A. Delga, H.-R. Ren, H.-D. Chen, C. Xu, P. Genevet, Metasurface-integrated vertical cavity surface-emitting lasers for programmable directional lasing emissions. *Nat. Nanotechnol.* **15**, 125–130 (2020).

13. H. Aouani, O. Mahboub, E. Devaux, H. Rigneault, T. W. Ebbesen, J. Wenger, Plasmonic antennas for directional sorting of fluorescence emission. *Nano Lett.* **11**, 2400–2406 (2011).

14. J. Zhou, M. Xia, Y. Chen, X. Zhang, Circular-polarization-dependent beam deflection via brillouin zone folding in resonant phase gradient metasurfaces. *ACS Photonics* **11**, 2707–2712 (2024).

15. J. Zhou, Y. Wang, M. Xia, Y. Chen, D. Huang, X. Zhang, Excitonic van der Waals metasurfaces for resonant wavefront shaping at deep subwavelength thickness scale. *Nano Lett.* **24**, 9658–9665 (2024).

16. M. Xia, Y. Chen, J. Zhou, Y. Wang, D. Huang, X. Zhang, Spin-locked WS_2 vortex emission via photonic crystal bound states in the continuum. *Adv. Mater.* **36**, 2400214 (2024).

17. J. Shang, C. Cong, Z. Wang, N. Peimyoo, L. Wu, C. Zou, Y. Chen, X. Y. Chin, J. Wang, C. Soci, W. Huang, T. Yu, Room-temperature 2D semiconductor activated vertical-cavity surface-emitting lasers. *Nat. Commun.* **8**, 543 (2017).

18. Y. Bu, X. Ren, J. Zhou, Z. Zhang, J. Deng, H. Xu, R. Xie, T. Li, W. Hu, X. Guo, W. Lu, X. Chen, Configurable circular-polarization-dependent optoelectronic silent state for ultrahigh light ellipticity discrimination. *Light Sci. Appl.* **12**, 176 (2023).

19. Y. Wang, D. Huang, M. Xia, J. Zhou, Y. Chen, X. Zhang, Polarization-controlled exciton-polaritons in WS_2 strongly coupled with low-symmetry photonic crystal nanostructures. *Nano Lett.* **24**, 11551–11558 (2024).

20. A. F. Cihan, A. G. Curto, S. Raza, P. G. Kik, M. L. Brongersma, Silicon Mie resonators for highly directional light emission from monolayer MoS_2. *Nat. Photonics* **12**, 284–290 (2018).

21. X. Zhang, S. Choi, D. Wang, C. H. Naylor, A. T. C. Johnson, E. Cubukcu, Unidirectional doubly enhanced MoS_2 emission via photonic fano resonances. *Nano Lett.* **17**, 6715–6720 (2017).

22. J. Wang, H. Li, Y. Ma, M. Zhao, W. Liu, B. Wang, S. Wu, X. Liu, L. Shi, T. Jiang, J. Zi, Routing valley exciton emission of a WS_2 monolayer via delocalized Bloch modes of in-plane inversion-symmetry-broken photonic crystal slabs. *Light Sci. Appl.* **9**, 148 (2020).

23. S. Li, R. Ai, K. K. Chui, Y. Fang, Y. Lai, X. Zhuo, L. Shao, J. Wang, H.-Q. Lin, Routing the exciton emissions of WS_2 monolayer with the high-order plasmon modes of Ag nanorods. *Nano Lett.* **23**, 4183–4190 (2023).

24. X. Zhang, C. De-Eknamkul, J. Gu, A. L. Boehmke, V. M. Menon, J. Khurgin, E. Cubukcu, Guiding of visible photons at the ångström thickness limit. *Nat. Nanotechnol.* **14**, 844–850 (2019).

25. X. Zhang, N. Biekert, S. Choi, C. H. Naylor, C. De-Eknamkul, W. Huang, X. Zhang, X. Zheng, D. Wang, A. T. C. Johnson, E. Cubukcu, Dynamic photochemical and optoelectronic control of photonic fano resonances via monolayer MoS_2 trions. *Nano Lett.* **18**, 957–963 (2018).

26. X. Sun, Y. Zhu, H. Qin, B. Liu, Y. Tang, T. Lü, S. Rahman, T. Yildirim, Y. Lu, Enhanced interactions of interlayer excitons in free-standing heterobilayers. *Nature* **610**, 478–484 (2022).

27. J. Zhou, D. Huang, Y. Wang, Y. Chen, M. Xia, X. Zhang, Chiral absorption enhancement via critically coupled resonances in atomically thin photonic crystal exciton-polaritons. *Opt. Lett.* **49**, 3990–3993 (2024).

28. Y. Wang, M. Xia, J. Zhou, D. Huang, Y. Chen, X. Zhang, Resonantly enhanced optical birefringence in ultrathin high-index WS_2 metasurfaces. *Laser Photonics Rev.* **18**, 2301088 (2024).

29. L. Yuan, B. Zheng, J. Kunstmann, T. Brumme, A. B. Kuc, C. Ma, S. Deng, D. Blach, A. Pan, L. Huang, Twist-angle-dependent interlayer exciton diffusion in WS_2–WSe_2 heterobilayers. *Nat. Mater.* **19**, 617–623 (2020).

30. H. Chen, X. Wen, J. Zhang, T. Wu, Y. Gong, X. Zhang, J. Yuan, C. Yi, J. Lou, P. M. Ajayan, W. Zhuang, G. Zhang, J. Zheng, Ultrafast formation of interlayer hot excitons in atomically thin MoS_2/WS_2 heterostructures. *Nat. Commun.* **7**, 12512 (2016).

31. A. Raja, A. Chaves, J. Yu, G. Arefe, H. M. Hill, A. F. Rigosi, T. C. Berkelbach, P. Nagler, C. Schüller, T. Korn, C. Nuckolls, J. Hone, L. E. Brus, T. F. Heinz, D. R. Reichman, A. Chernikov, Coulomb engineering of the bandgap and excitons in two-dimensional materials. *Nat. Commun.* **8**, 15251 (2017).

32. P. K. Nayak, Y. Horbatenko, S. Ahn, G. Kim, J.-U. Lee, K. Y. Ma, A.-R. Jang, H. Lim, D. Kim, S. Ryu, H. Cheong, N. Park, H. S. Shin, Probing evolution of twist-angle-dependent interlayer excitons in $MoSe_2$/WSe_2 van der Waals heterostructures. *ACS Nano* **11**, 4041–4050 (2017).

33. X. Zhang, X. Zhang, W. Huang, K. Wu, M. Zhao, A. T. Charlie Johnson, S. Tongay, E. Cubukcu, Ultrathin WS_2-on-glass photonic crystal for self-resonant exciton-polaritonics. *Adv. Opt. Mater.* **8**, 1901988 (2020).

34. H. Yu, Y. Wang, Q. Tong, X. Xu, W. Yao, Anomalous light cones and valley optical selection rules of interlayer excitons in twisted heterobilayers. *Phys. Rev. Lett.* **115**, 187002 (2015).

35. J. S. Ross, P. Rivera, J. Schaibley, E. Lee-Wong, H. Yu, T. Taniguchi, K. Watanabe, J. Yan, D. Mandrus, D. Cobden, W. Yao, X. Xu, Interlayer exciton optoelectronics in a 2D heterostructure p-n junction. *Nano Lett.* **17**, 638–643 (2017).

36. Y. Jiang, S. Chen, W. Zheng, B. Zheng, A. Pan, Interlayer exciton formation, relaxation, and transport in TMD van der Waals heterostructures. *Light Sci. Appl.* **10**, 72 (2021).

37. M. Luo, Y. Zhou, X. Zhao, Z. Guo, Y. Li, Q. Wang, J. Liu, W. Luo, Y. Shi, A. Q. Liu, X. Wu, High-sensitivity optical sensors empowered by quasi-bound states in the continuum in a hybrid metal-dielectric metasurface. *ACS Nano* **18**, 6477–6486 (2024).

38. Y. Chen, H. Deng, X. Sha, W. Chen, R. Wang, Y.-H. Chen, D. Wu, J. Chu, Y. S. Kivshar, S. Xiao, C.-W. Qiu, Observation of intrinsic chiral bound states in the continuum. *Nature* **613**, 474–478 (2023).

39. D. Huang, J. Choi, C.-K. Shih, X. Li, Excitons in semiconductor moiré superlattices. *Nat. Nanotechnol.* **17**, 227–238 (2022).

40. Z. Wang, M. Sebek, X. Liang, A. Elbanna, A. Nemati, N. Zhang, C. H. K. Goh, M. Jiang, J. Pan, Z. Shen, X. Su, N. T. K. Thanh, H. Sun, J. Teng, Greatly enhanced resonant exciton-trion conversion in electrically modulated atomically thin WS_2 at room temperature. *Adv. Mater.* **35**, 2302248 (2023).

41. G. Cheng, B. Li, Z. Jin, M. Zhang, J. Wang, Observation of diffusion and drift of the negative trions in monolayer WS_2. *Nano Lett.* **21**, 6314–6320 (2021).

42. H.-L. Liu, T. Yang, J.-H. Chen, H.-W. Chen, H. Guo, R. Saito, M.-Y. Li, L.-J. Li, Temperature-dependent optical constants of monolayer MoS_2 , $MoSe_2$, WS_2 , and WSe_2: Spectroscopic ellipsometry and first-principles calculations. *Sci. Rep.* **10**, 15282 (2020).

43. Y. Zhong, L. Zhang, J.-H. Park, S. Cruz, L. Li, L. Guo, J. Kong, E. N. Wang, A unified approach and descriptor for the thermal expansion of two-dimensional transition metal dichalcogenide monolayers. *Sci. Adv.* **8**, eabo3783 (2022).

44. X. Zhang, W. Huang, C. De-Eknamkul, K. Wu, M.-Q. Zhao, S. Tongay, A. T. Charlie Johnson, E. Cubukcu, Azimuthally polarized and unidirectional excitonic emission from deep subwavelength transition metal dichalcogenide annular heterostructures. *ACS Photonics* **8**, 2861–2867 (2021).

45. Y. Wang, D. Huang, M. Xia, J. Zhou, Y. Chen, Y. Liao, X. Zhang, Directional chiral exciton emission via topological polarization singularities in all van der Waals metasurfaces. *Adv. Mater.* **37**, 2414174 (2024).
